# Associations of four insulin resistance indicators with subsequent pregnancy outcomes in women with recurrent pregnancy loss

**DOI:** 10.3389/fendo.2026.1767301

**Published:** 2026-04-02

**Authors:** Mei Wang, Fangxiang Mu, Fang Wang

**Affiliations:** 1 Obstetrics and Gynaecology Intensive Care Unit, Gansu Provincial Maternity and Child Health Hospital (Gansu Provincial Central Hospital), Lanzhou, Gansu, China; 2 Department of Reproductive Medicine, Lanzhou University Second Hospital, Lanzhou, Gansu, China

**Keywords:** cohort study, insulin resistance, METS-IR, recurrent miscarriage, recurrent pregnancy loss, TyG-BMI

## Abstract

**Aims:**

This study investigated the associations of four insulin resistance (IR) indicators—the triglyceride-glucose (TyG) index, the TyG-body mass index (TyG-BMI), the metabolic score for IR (METS-IR), and the triglyceride to high-density lipoprotein cholesterol (TG/HDL-C) ratio—with subsequent pregnancy outcomes in women with recurrent pregnancy loss (RPL) and assessed their predictive value.

**Methods:**

This cohort study recruited RPL participants from the Chinese Pregnancy Loss Cohort. Enrollment occurred between September 2019 and December 2022. All participants were followed up every 6 months, with a minimum follow-up duration of 18 months, to document pregnancy outcomes (live birth or subsequent pregnancy loss). Univariate and multivariate logistic regression analyses were performed to assess the associations between four IR indicators (TG/HDL-C, TyG, TyG-BMI, METS-IR) and pregnancy outcomes. Receiver operating characteristic (ROC) analysis was conducted to determine the predictive efficacy of each indicator.

**Results:**

Among 2,454 screened participants, 897 RPL women were analyzed (638 live births, 71.1%; 259 pregnancy losses, 28.9%). In the fully adjusted model, the highest tertiles of TyG-BMI and METS-IR were associated with significantly elevated odds of pregnancy loss (OR = 1.52, 95% CI: 1.01–2.27, *P* = 0.044; OR = 1.49, 95% CI: 1.05–2.29, *P* = 0.045, respectively). METS-IR demonstrated the highest predictive efficacy for pregnancy outcomes (AUC = 0.710), followed by TyG-BMI, TG/HDL-C, and the TyG index.

**Conclusions:**

Among women with RPL, TyG-BMI and METS-IR are independently associated with increased pregnancy loss risk, with METS-IR demonstrating superior predictive performance.

## 1 Introduction

Recurrent pregnancy loss (RPL) is defined as two or more spontaneous pregnancy losses before 24 weeks of gestation (1). The etiology of RPL is complex and involves genetic predispositions, anatomical anomalies, endocrine disorders, immune dysfunction, and infectious agents (2). However, approximately 50% of RPL cases are classified as idiopathic, lacking a definitive etiological diagnosis or targeted intervention strategies, which poses a significant clinical challenge for reproductive-aged women worldwide (3).

Insulin resistance (IR), a prevalent metabolic disorder characterized by diminished sensitivity of target tissues to insulin, has gained recognition as a potential underlying pathogenic factor in unexplained recurrent pregnancy loss (URPL) (4). IR has been definitively linked to reproduction-related abnormalities, such as polycystic ovarian syndrome (PCOS), endometrial dysfunction (5), and compromised oocyte quality (6), each of which significantly contributes to adverse pregnancy outcomes.

The preconception period is a critical window for intervening in metabolic abnormalities and optimizing pregnancy outcomes (7, 8). However, current studies on whether IR levels during this crucial window influence subsequent pregnancy outcomes in patients with RPL remain scarce. Various methods are available for assessing insulin resistance, among which the Homeostasis Model Assessment of Insulin Resistance (HOMA-IR) is widely used due to its simplicity requiring only fasting glucose and insulin levels. However, HOMA-IR cut-off values vary significantly across ethnicities, sexes, and ages. For instance, the 75th percentile values range from 1.6 in Iranians to 2.53 in Koreans, 2.0 in Swedish men, and 3.8 in French men (9). Among Asian populations, the 90th percentile was 1.7 in Japanese (10), while ROC-derived optimal cut-offs were 2.39-2.48 for Korean women (9), and 1.4-2.0 for Southern Chinese (11). Given these variations, the present study adopted a cut-off value of 2.5 based on previous studies in RPL patients as the diagnostic criterion for HOMA-IR (12).

As an indirect method, the measurement of IR indicators is derived from readily available and cost-effective clinical parameters such as blood glucose, triglycerides, and body mass index (BMI), without additional insulin testing. Common IR indicators include the triglyceride-glucose (TyG) index (13), TyG-body mass index (TyG-BMI) (14), metabolic score for insulin resistance (METS-IR) (15), and triglyceride-high-density lipoprotein cholesterol (TG/HDL) ratio (16). Although these IR indicators have been used to evaluate IR status in various populations (17–20). Whether they can effectively identify pre-pregnant RPL women at high risk of pregnancy loss has not been fully verified. Therefore, this study aims to enroll a prospective cohort of women with RPL to examine the association between the value of four pre-conception IR-related indicators (TyG index, TyG-BMI, METS-IR, and TG/HDL-C) and the risk of subsequent pregnancy outcomes.

## 2 Materials and methods

### 2.1 Data source

Data for this study were extracted from the Pregnancy Loss Cohort of the Real-World Study on Recurrent Pregnancy Loss in China (Project number: YJS-BD-19). The cohort is registered in the Chinese Clinical Trial Registry (registration number: ChiCTR2000039414). This study was approved by the Ethics Committee of Lanzhou University Second Hospital (approval number: 2019A-231).

### 2.2 Participants and selection criteria

Between September 2019 and December 2022, we enrolled women with one or more prior pregnancy losses into the cohort. All participants provided written informed consent at enrollment, and information on potential etiologies, risk factors, and medical examinations was collected at recruitment. The inclusion criteria were a history of two or more pregnancy losses, age ranging from 18 to 42 years, and a clear intention to pursue future fertility, while the exclusion criteria included pregnancy loss caused by a single confirmed, corrected or irreversible etiology, a history of PCOS, a confirmed diagnosis of infertility, incomplete key medical record data precluding comprehensive analysis, and severe mental illness that prevents voluntary signing of informed consent for follow-up.

After the initial consultation, detailed disease histories were recorded for each participant, including age, ethnicity, education, height, weight, pregnancy losses, menstrual history, and other medical details. A comprehensive physical exam and uterine imaging were then conducted. Participants were monitored every six months for at least 18 months, ending by August 2024.

Blood samples were taken after an 8-hour fast and analyzed within 3 hours. Fasting blood glucose (FBG), fasting insulin (INS), fasting C-peptide (FCP), 2-hour postprandial blood glucose (2hPBG), 2-hour postprandial insulin (2hINS), 2-hour postprandial C-peptide (2hCP), and 25−hydroxyvitamin D [25(OH)D] were measured by chemiluminescent immunoassay (Roche Diagnostics GmbH, Mannheim, Germany). Furthermore, levels of total cholesterol (TC), triglycerides (TG), high-density lipoprotein cholesterol (HDL-C), low-density lipoprotein cholesterol (LDL-C), uric acid (UA), total bilirubin (TBIL), direct bilirubin (DBIL), and indirect bilirubin (IBIL), alanine aminotransferase (ALT), aspartate aminotransferase (AST), and homocysteine (HCY) were quantified using the Beckman Coulter AU5800. Serum thyroid function indicators, including thyroid-stimulating hormone (TSH), free triiodothyronine (FT3), and free thyroxine (FT4), were quantified using an enzyme-linked immunosorbent assay (Roche Diagnostics GmbH, Mannheim, Germany).

### 2.3 Pregnancy outcomes

Women with RPL who became pregnant again after their initial visit experienced two outcomes: pregnancy loss and live birth. Pregnancy loss is defined as spontaneous pregnancy loss before 24 weeks, while live birth refers to the survival of the fetus at or after 24 weeks of gestation.

### 2.4 Definition of variables

In this study, IR was assessed based on the HOMA-IR, which is derived from fasting plasma glucose and fasting insulin concentrations. IR was diagnosed by the following indicators (1): impaired glucose tolerance (IGT) or diabetes mellitus (21) (2); fasting insulin levels (FINS) ≥15 mIU/L (22) (3); HOMA-IR = [FPG (mmol/L) × INS (mIU/L)]/22.5 > 2.5 (23).

Four indicators of IR were calculated using the following equations:


$$TyG\;=\;ln\;\left[TG\;\left(mg/dL\right)\;\times \;FBG\;\left(mg/dL\right)/2\right];$$

$$METS-IR\;=\;ln\;\left[2\times \;FPG\;\left(mg/dL\right)\;+\;TG\;\left(mg/dL\right)\right]\;\times \;BMI\;\left(kg/m^{2}\right)/ln\;\left[HDL-C\;\left(mg/dL\right)\right];$$

$$TyG-BMI\;=\;TyG\;\times \;BMI\;\left(kg/m^{2}\right);\;TG/HDL-C\;=\;TG\;\left(mg/dL\right)/HDL-C\;\left(mg/dL\right).$$

Laboratory measurements of FBG, TG, and HDL-C, initially recorded in mmol/L, were converted to mg/dL using the following conversion factors: HDL-C (mg/dL) = HDL-C (mmol/L) × 38.7; FBG (mg/dL) = FBG (mmol/L) × 18.0; TG (mg/dL) = TG (mmol/L) × 88.5.

### 2.5 Statistical analysis

Categorical variables were represented as percentages, while continuous variables with a normal distribution were expressed as mean ± standard deviation. Group comparisons were conducted using the independent samples t-test. For continuous variables with a skewed distribution, data were presented as median and interquartile ranges (IQR). Categorical data were also presented as counts with corresponding percentages. Missing data were addressed through multiple imputations. To investigate potential associations between four IR indicators and the HOMA-IR, a generalized additive model (GAM) with smooth curve fitting was employed. Optimal cutoff values were identified using the maximum Youden index. Univariate and multivariate logistic regression analyses were conducted to examine the associations between the four IR-related indicators and subsequent pregnancy outcomes, with results reported as odds ratios (ORs) and 95% confidence intervals (CIs). Additionally, receiver operating characteristic (ROC) curve analysis was performed to evaluate the discriminative ability of the TyG index, TyG-BMI, TG/HDL-C, and METS-IR in predicting pregnancy outcomes. Stratified sensitivity analyses were conducted according to age, number of pregnancy losses, and insulin resistance treatment status. All statistical analyses were performed using EmpowerStats software version 5.2 (https://www.empowerstats.com, X&Y Solutions, Inc., Boston, MA) and R language (http://www.R-project.org).

## 3 Results

### 3.1 Baseline characteristics

**Figure 1:**
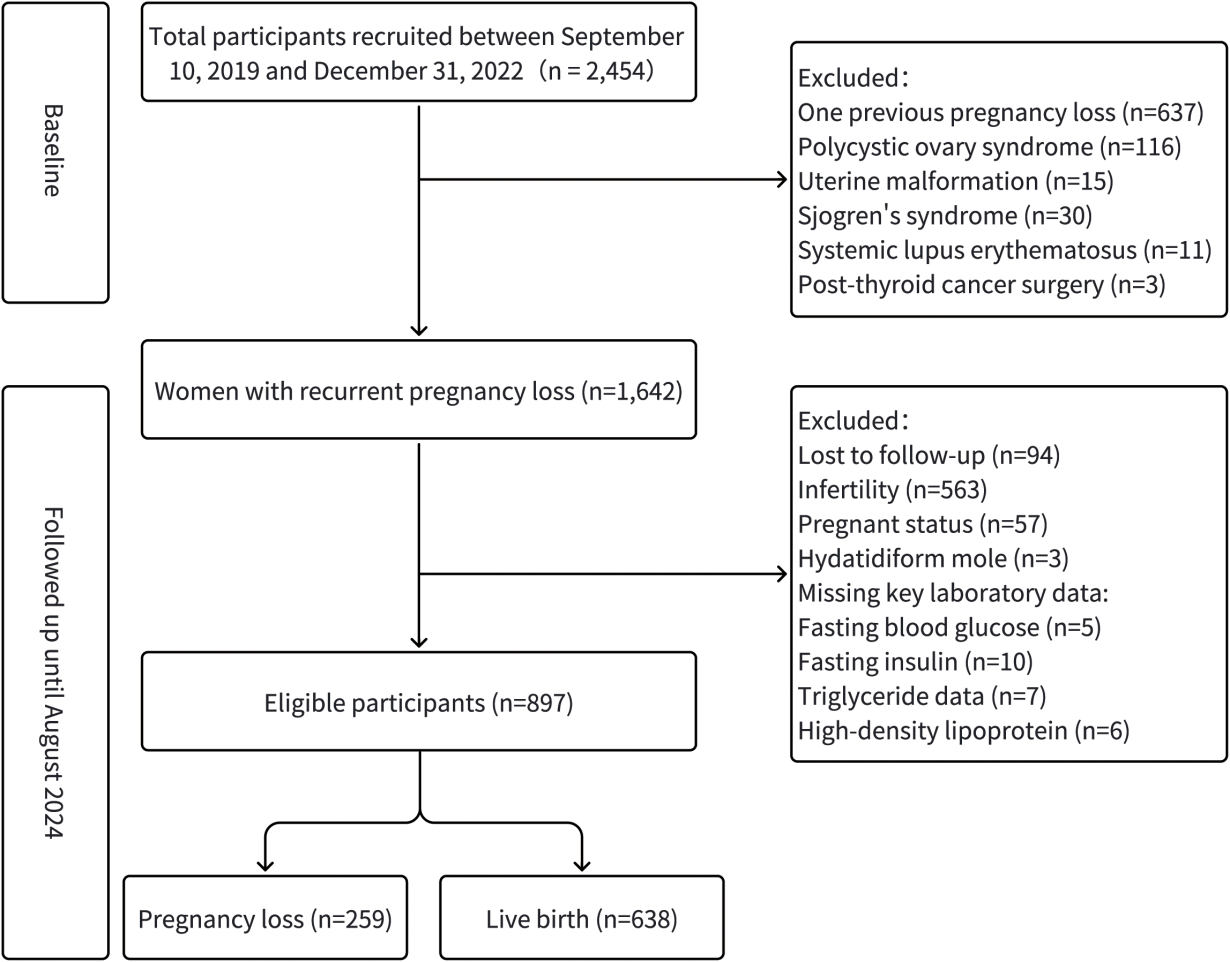
Flowchart of participant enrollment and follow-up in women with recurrent pregnancy loss.

A total of 2,454 women were recruited between September 10, 2019, and December 31, 2022. After excluding 812 individuals (due to one previous pregnancy loss, polycystic ovary syndrome, uterine malformation, or other comorbidities), 1,642 women with recurrent pregnancy loss were identified. During follow-up (until August 2024), an additional 745 participants were excluded (including loss to follow-up, infertility, missing key laboratory data, etc.), resulting in 897 eligible participants (**Figure 1**). Within this cohort, 638(71.1%) participants achieved a live birth outcome, while 259(28.9%) experienced subsequent pregnancy loss.

A total of 897 patients with RPL were classified into two groups: the non-IR group (n= 472, 52.6%) and the IR group (n=425, 47.4%) (**Table 1**). Age showed a significant between-group difference (*P* = 0.005), with no significant differences observed in other demographic and reproductive baseline characteristics, including educational level, ethnicity, menstrual status, pregnancy loss type, and number of previous pregnancy losses (all *P* > 0.05). The clinical intervention rate differed significantly between the two groups (*P*< 0.001): no participants in the non-IR group received intervention, while 60.47% (257/425) of the IR group underwent clinical intervention.

**Table 1:** Baseline clinical and biochemical characteristics of participants.

Parameters	Non-IR group	IR group	*P*-value
N	472	425	
Age(years)	30.07 ± 4.06	30.86 ± 4.29	0.005
BMI (kg/m^2^)	21.61 ± 2.55	23.33 ± 3.21	<0.001
Education (n, %)			0.440
Primary School	29 (6.14)	18 (4.24)	
High School	128 (27.12)	117 (27.53)	
College	315 (66.74)	290 (68.24)	
Race (n, %)			0.962
Han nationality	422 (89.41)	382 (89.88)	
Hui nationality	31 (6.57)	26 (6.12)	
Other minority nationality	19 (4.03)	17 (4.00)	
Menstrual cycles (n, %)			0.529
Regular	410 (86.86)	363 (85.41)	
Irregular	62 (13.14)	62 (14.59)	
Pregnancy loss types (n, %)			0.963
Primary	367 (77.75)	331 (77.88)	
Secondary	105 (22.25)	94 (22.12)	
Previous pregnancy losses (n, %)			0.713
2 times	304 (64.41)	272 (64.00)	
3 times	118 (25.00)	101 (23.76)	
≥ 4 times	50 (10.59)	52 (12.24)	
Clinical intervention (n, %)			<0.001
No	472 (100.00)	168 (39.53)	
Yes	0 (0.00)	257 (60.47)	
25(OH)D (nmol/L)	12.82 ± 5.26	12.95 ± 4.85	0.713
FT3 (pmol/L)	5.21 ± 0.58	5.17 ± 1.13	0.501
FT4 (pmol/L)	16.22 ± 2.53	16.08 ± 3.18	0.490
TSH (mIU/L)	2.43 (1.70-3.10)	2.46 (1.78-3.17)	0.140
HCY (μmol/L)	11.50 ± 4.16	11.18 ± 4.18	0.248
FPG (mmol/L)	4.84 ± 0.46	5.31 ± 1.04	<0.001
INS (mIU/L)	7.38 (5.71-9.11)	14.64 (12.34-18.99)	<0.001
FCP (ng/ml)	1.05 (0.87-1.28)	1.74 (1.46-2.16)	<0.001
2hPG (mmol/L)	5.54 (4.93-6.20)	6.40 (5.63-7.61)	<0.001
2hINS (mIU/L)	30.49 (20.93-45.38)	58.45 (36.51-86.84)	<0.001
2hCP (ng/ml)	3.82 (2.98-5.10)	5.83 (4.16-7.70)	<0.001
TC (mmol/L)	3.90 ± 0.73	4.06 ± 0.76	0.002
TG (mmol/L)	1.02 ± 0.53	1.34 ± 0.79	<0.001
HDL-C (mmol/L)	1.40 ± 0.34	1.31 ± 0.36	<0.001
LDL-C (mmol/L)	2.41 ± 0.58	2.59 ± 0.68	<0.001
Cr (μmol/L)	53.12 ± 7.78	53.25 ± 8.22	0.808
UA (μmol/L)	254.93 ± 57.08	275.55 ± 67.34	<0.001
TBIL (μmol/L)	12.83 ± 5.36	12.47 ± 4.80	0.290
DBIL (μmol/L)	3.11 ± 2.24	2.75 ± 1.69	0.008
IBIL (μmol/L)	9.69 ± 4.49	9.69 ± 4.38	0.986
ALT(U/L)	14.00 (10.00-19.00)	18.00 (12.00-26.00)	<0.001
AST(U/L)	20.00 (17.00-23.09)	21.46 (18.00-26.00)	<0.001
TyG index	8.17 ± 0.44	8.51 ± 0.51	<0.001
TG/HDL-C	1.80 ± 1.25	2.61 ± 1.98	<0.001
TyG-BMI	176.70 ± 24.41	198.82 ± 32.03	<0.001
METS-IR	30.46 ± 4.68	34.47 ± 5.99	<0.001

Data are shown as mean ± standard deviation, median(min-max) or frequency with percentages.

IR, insulin resistance; BMI, body mass index; 25(OH)D, 25-hydroxyvitamin D; FT3, free triiodothyronine; FT4, free thyroxine; TSH, thyroid-stimulating hormone; HCY, homocysteine; INS, insulin; FPG, fasting plasma glucose; FCP, fasting C-peptide; 2hINS, 2-hour postprandial insulin; 2hPG, 2-hour postprandial plasma glucose; 2hCP, 2-hour postprandial C-peptide; TC, total cholesterol; HDL-C, high-density lipoprotein cholesterol; LDL-C, low-density lipoprotein cholesterol; UA, uric acid; TBIL, total bilirubin; DBIL, direct bilirubin; IBIL, indirect bilirubin; ALT, alanine transaminase; AST, aspartate transaminase; TyG, triglyceride-glucose index; TG/HDL-C, triglyceride/high-density lipoprotein cholesterol; TyG-BMI, triglyceride-glucose body mass index; RPL, recurrent pregnancy loss; IR, insulin resistance; METS-IR, metabolic score for insulin resistance.

In contrast, compared with the non-IR group, the IR group exhibited significantly elevated levels of BMI, FBG,2hPBG, INS, FCP, TC, TG, LDL-C, UA, ALT, AST, and all IR-related indicators (TyG index, TyG-BMI, METS-IR, and TG/HDL-C) (all *P*< 0.05). Meanwhile, the IR group had significantly lower levels of HDL-C (*P*< 0.001) and DBIL (*P* = 0.008). No significant differences in 25(OH)D, thyroid function indicators (TSH, FT3, FT4), HCY, Cr, TBIL, and IBIL levels were observed between the two groups (all *P* > 0.05).

### 3.2 The association between four IR Indicators and IR

**Figure 2:**
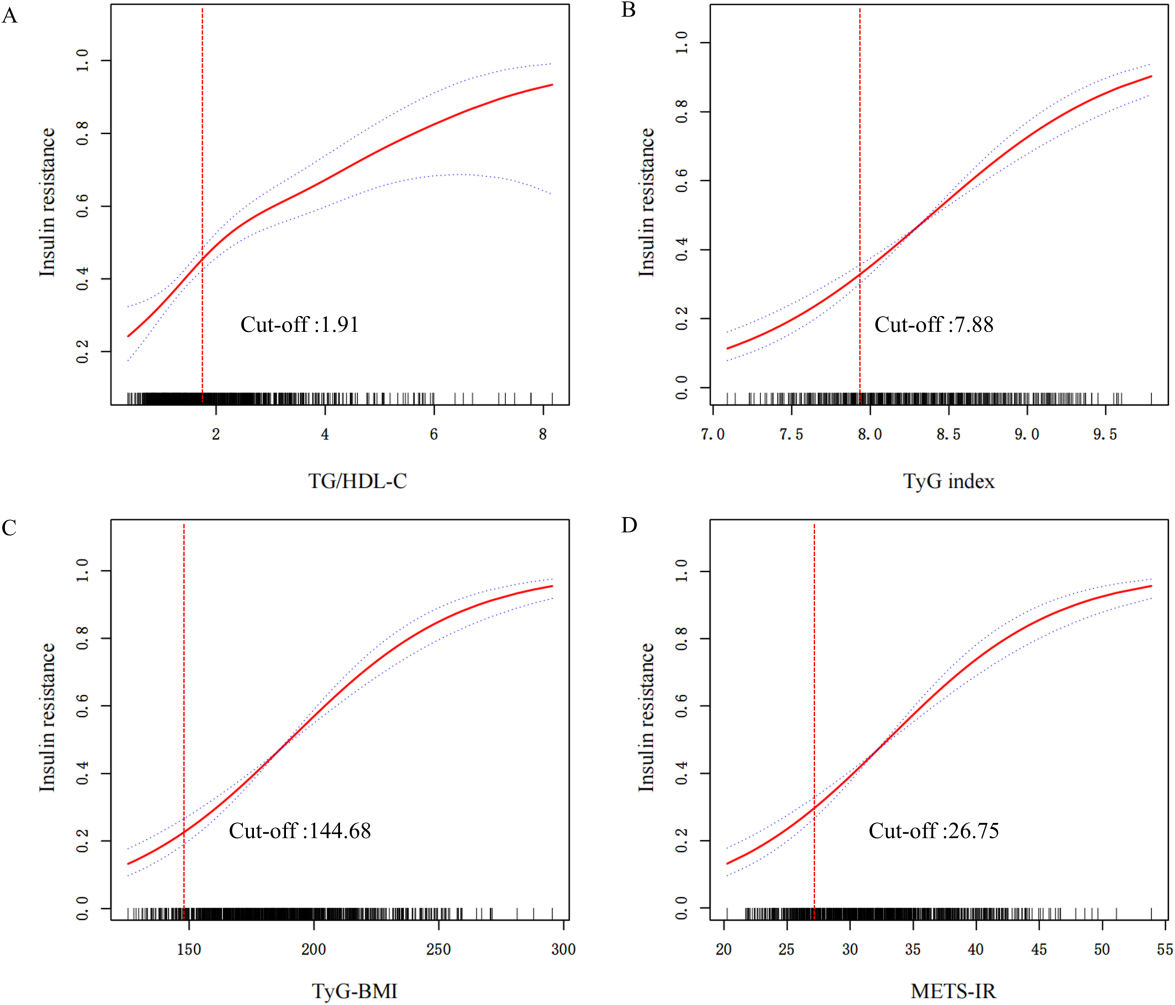
Smooth curve fitting and threshold effect analysis of four IR indicators and IR **(A)** TG/HDL-C index (cut-off: 1.91); **(B)** TyG index (cut-off: 7.88); **(C)** TyG-BMI index (cut-off: 144.68); **(D)** METS-IR index (cut-off: 26.75). The solid red line represents the ROC curve, the dashed blue lines represent the 95% confidence interval, and the red dashed vertical line indicates the optimal cut-off value for each index.

Restricted cubic spline (RCS) analysis with smooth curve fitting revealed nonlinear increasing associations of TG/HDL-C ratio, TyG-BMI, and METS-IR with HOMA-IR, whereas the TyG index showed a linear increasing trend. As shown in **Figure 2**, threshold effect analysis identified cutoff values of 1.91 for TG/HDL-C ratio (**Figure 2A**), 7.88 for TyG index (**Figure 2B**), 144.68 for TyG-BMI (**Figure 2C**), and 26.75 for METS-IR (**Figure 2D**).

### 3.3 Univariate analysis of subsequent pregnancy outcomes

A positive correlation was identified between age, BMI, a history of ≥ 4 pregnancy losses, TG, TG/HDL-C, TyG-BMI, and METS-IR and the risk of pregnancy loss, with all associations reaching statistical significance (all *P*< 0.05). The TyG index was marginally associated with pregnancy loss risk (*P* = 0.057). Higher HDL-C levels were linked to a lower risk of pregnancy loss (OR = 0.62, 95% CI: 0.40–0.97, *P* = 0.035). No significant correlations were found with educational level, ethnicity, menstrual cycle, type of pregnancy loss, hyperuricemia, and hyperhomocysteinemia (all *P*>0.05). All these results are summarized in **Table 2**.

**Table 2:** Univariate associations between variables and subsequent pregnancy loss.

	Subsequent pregnancy outcomes		
Parameters	N	OR (95%CI)	*P-value*
Age(years)	30 ± 4	1.06 (1.02, 1.10)	0.001
BMI (kg/m^2^)	22.43 ± 3.00	1.06 (1.01, 1.11)	0.022
Education (n, %)			
Primary School	47 (5.24)	1.0	
High School	245 (27.31)	0.71 (0.37, 1.36)	0.302
College	605 (67.45)	0.61 (0.33, 1.13)	0.114
Race (n, %)			
Han nationality	804 (89.63)	1.0	
Hui nationality	57 (6.35)	0.94 (0.52, 1.72)	0.852
Other nationality	36 (4.01)	0.69 (0.31, 1.54)	0.367
Menstrual cycles (n, %)			
Regular	773 (86.18)	1.0	
Irregular	124 (13.82)	1.20 (0.80, 1.81)	0.371
Pregnancy loss types (n, %)			
Primary	698 (77.81)	1.0	
Secondary	199 (22.19)	1.12 (0.79, 1.57)	0.530
Previous pregnancy losses (n, %)			
2 times	576 (64.21)	1.0	
3 times	219 (24.41)	1.21 (0.86, 1.71)	0.267
≥ 4 times	102 (11.37)	1.97 (1.27, 3.05)	0.002
Clinical intervention			
No	640 (71.35%)	1.0	
Yes	257 (28.65%)	0.89 (0.65, 1.23)	0.493
25(OH)D (nmol/L)	12.88 ± 5.06	1.00 (0.97, 1.03)	0.967
FT3 (pmol/L)	5.19 ± 0.88	0.72 (0.56, 0.94)	0.013
FT4 (pmol/L)	16.15 ± 2.85	1.00 (0.95, 1.05)	0.982
TSH (mIU/L)	2.88 ± 2.73	1.01 (0.96, 1.06)	0.709
HCY (μmol/L)	11.35 ± 4.17	0.99 (0.96, 1.03)	0.657
FPG (mmol/L)	5.05 ± 0.72	0.93 (0.74, 1.15)	0.485
INS (IU/L)	11.83 ± 6.89	0.99 (0.97, 1.01)	0.349
FCP (ng/ml)	1.50 ± 0.85	1.02 (0.86, 1.21)	0.823
2hPG (mmol/L)	6.23 ± 1.84	1.05 (0.97, 1.14)	0.195
2hINS (IU/L)	51.28 ± 40.63	1.00 (1.00, 1.01)	0.344
2hCP (ng/ml)	5.13 ± 2.52	1.04 (0.98, 1.10)	0.225
TC (mmol/L)	3.98 ± 0.76	1.12 (0.92, 1.35)	0.253
TG (mmol/L)	1.19 ± 0.85	1.28 (1.06, 1.54)	0.011
HDL-C (mmol/L)	1.36 ± 0.35	0.62 (0.40, 0.97)	0.035
LDL-C (mmol/L)	2.49 ± 0.64	1.25 (1.00, 1.57)	0.050
Cr (μmol/L)	53.19 ± 7.99	0.99 (0.98, 1.01)	0.502
UA(μmol/L)	265.03 ± 64.09	1.00 (1.00, 1.00)	0.252
TBIL (μmol/L)	12.74 ± 5.36	1.00 (0.98, 1.03)	0.752
DBIL (μmol/L)	2.96 ± 2.08	0.98 (0.91, 1.05)	0.566
IBIL (μmol/L)	9.80 ± 4.68	1.01 (0.98, 1.04)	0.584
ALT(U/L)	19.51 ± 16.50	1.00 (0.99, 1.01)	0.786
AST(U/L)	22.95 ± 11.42	0.99 (0.98, 1.01)	0.248
TG/HDL-C	2.18 ± 1.68	1.13 (1.04, 1.23)	0.003
TyG index	8.33 ± 0.50	1.32 (0.99, 1.75)	0.057
TyG-BMI	187.18 ± 30.34	1.01 (1.00, 1.01)	0.009
METS-IR	32.36 ± 5.70	1.04 (1.01, 1.06)	0.006

Note: Data are shown as mean ± standard deviation, median(min-max) or frequency with percentages.

OR, dds ratio; 95% CI, 95% confidence interval. BMI, body mass index; 25(OH)D, 25-hydroxyvitamin D; FT3, free triiodothyronine; FT4, free thyroxine; TSH, thyroid-stimulating hormone; HCY, homocysteine; INS, insulin; FPG, fasting plasma glucose; FCP, fasting C-peptide; 2hINS, 2-hour postprandial insulin; 2hPG, 2-hour postprandial plasma glucose; 2hCP, 2-hour postprandial C-peptide; TC, total cholesterol; HDL-C, high-density lipoprotein cholesterol; LDL-C, low-density lipoprotein cholesterol; UA, uric acid; TBIL, total bilirubin; DBIL, direct bilirubin; IBIL, indirect bilirubin; ALT, alanine transaminase; AST, aspartate transaminase; TyG, triglyceride-glucose index; TG/HDL-C, triglyceride/high-density lipoprotein cholesterol; TyG-BMI, triglyceride-glucose body mass index; RPL, recurrent pregnancy loss; METS-IR, metabolic score for insulin resistance.

### 3.4 Association between four IR indicators and subsequent pregnancy outcomes

For evaluating the relationship between four IR indicators and pregnancy outcomes, we conducted a multivariate logistic regression analysis; participants were divided into three groups with 299 individuals in each group, with the low group serving as the reference for subsequent statistical comparisons. For TG/HDL-C: low group (≤ 1.33), middle group (1.33< value ≤ 2.28), and high group (>2.28); for TyG index: low group (≤ 6.76), middle group (6.76< value ≤ 7.16), and high group (>7.16); for TyG-BMI: low group (≤ 144.35), middle group (144.35< value ≤ 164.61), and high group (>164.61); for METS-IR: low group (≤ 25.79), middle group (25.79< value ≤ 30.17), and high group (>30.17).

Three models were used: Model I was an unadjusted model, Model II adjusted for age, and Model III adjusted for indicator-specific sets of covariates (detailed in the **Table 3** footnote). In the TG/HDL−C subgroup, the high group showed a significantly higher risk of pregnancy loss in Model I (OR = 1.44, 95% CI: 1.00–2.06, *P* = 0.048), but the association was not significant in Models II and III. The TyG index showed no significant association with pregnancy loss in any model. In contrast, the high TyG−BMI group consistently exhibited significantly elevated odds of pregnancy loss across all three models (Model I: OR = 1.61, 95% CI: 1.13–2.28, *P* = 0.008; Model II: OR = 1.45, 95% CI: 1.01–2.07, *P* = 0.045; Model III: OR = 1.52, 95% CI: 1.01–2.27, *P* = 0.044). Similarly, the high METS−IR group showed significantly increased odds of pregnancy loss in all models (Model I: OR = 1.53, 95% CI: 1.07–2.17, *P* = 0.018; Model II: OR = 1.41, 95% CI: 1.04–2.02, *P* = 0.038; Model III: OR = 1.49, 95% CI: 1.05–2.29, *P* = 0.045). No significant associations were observed in the middle groups for any of the four IR indicators. These results are presented in **Table 3**.

**Table 3:** Association between four IR indicators and subsequent pregnancy outcomes.

Exposure	Model I OR95%CI *P*-value	Model II OR95%CI *P*-value	Model III OR95%CI *P*-value
TG/HDL-C	1.13 (1.04, 1.23) 0.003	1.11 (1.02, 1.21) 0.012	1.13 (1.03, 1.24) 0.007
Low	Reference	Reference	Reference
Middle	1.43 (1.00, 2.05) 0.052	1.38 (0.96, 1.98) 0.085	1.31 (0.90, 1.92) 0.151
High	1.44 (1.00, 2.06) 0.048	1.32 (0.92, 1.90) 0.137	1.26 (0.86, 1.90) 0.223
TyG index	1.32 (0.99, 1.75) 0.057	1.20 (0.89, 1.60) 0.224	1.12 (0.80, 1.58) 0.514
Low	Reference	Reference	Reference
Middle	0.96 (0.67, 1.37) 0.815	0.91 (0.64, 1.31) 0.631	0.88 (0.60, 1.28) 0.500
High	1.19 (0.84, 1.69) 0.334	1.06 (0.74, 1.52) 0.759	0.88 (0.58, 1.34) 0.554
TyG-BMI	1.02 (1.02, 1.03) 0.001	1.02 (1.01, 1.03) 0.001	1.02 (1.01, 1.03) 0.001
Low	Reference	Reference	Reference
Middle	0.97 (0.67, 1.40) 0.851	0.93 (0.64, 1.35) 0.701	0.92 (0.63, 1.35) 0.673
High	1.61 (1.13, 2.28) 0.008	1.45 (1.01, 2.07) 0.045	1.52 (1.01, 2.27) 0.044
METS-IR	1.10 (1.06, 1.14) 0.001	1.10 (1.06, 1.14) 0.001	1.07 (1.02, 1.11) 0.006
Low	Reference	Reference	Reference
Middle	0.96 (0.66, 1.38) 0.814	0.93 (0.63, 1.32) 0.639	0.96 (0.65, 1.39) 0.804
High	1.53 (1.07, 2.17) 0.018	1.41 (1.04, 2.02) 0.038	1.49 (1.05, 2.29) 0.045

OR dds ratio; 95% CI = 95% confidence interval.

Model I: unadjusted model. Model II: adjusted for age.Model III: TyG index adjusted for age, FT3, INS, 2hPG, 2hCP, TC, LDL-C and Previous pregnancy losses. TG/HDL-C adjusted for age, FT3, INS, LDL-C and Previous pregnancy losses. TyG-BMI adjusted for age, INS FCP LDL-C and Previous pregnancy losses. METS-IR adjusted for age, INS FCP TC LDL-C and Previous pregnancy losses.

### 3.5 The four IR indicators predict subsequent pregnancy loss

**Figure 3:**
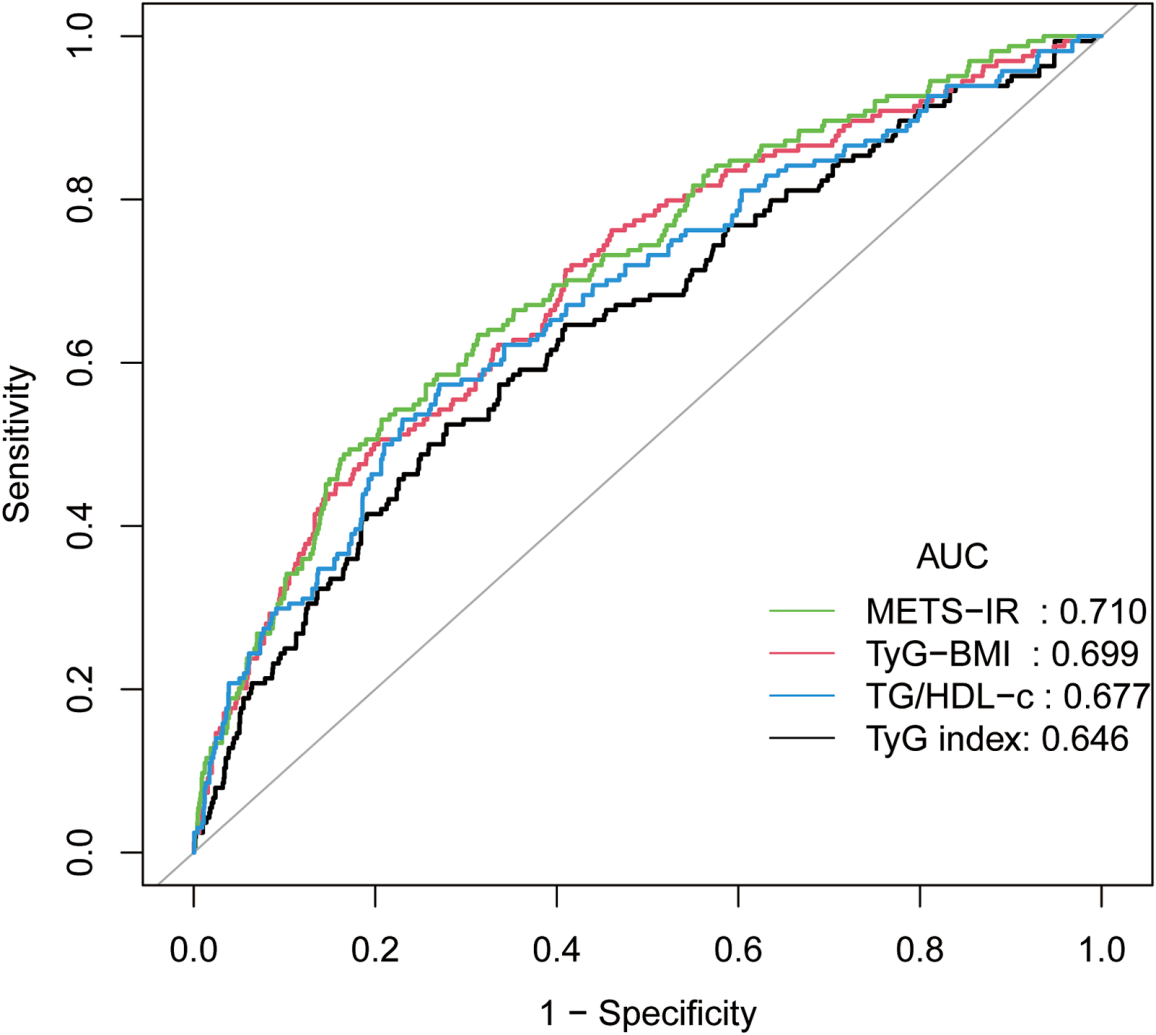
The four IR indicators predict subsequent pregnancy outcomes. Receiver operating characteristic (ROC) curve analysis of four insulin resistance (IR) indicators for predicting subsequent pregnancy loss in patients with recurrent pregnancy loss (RPL). Pregnancy outcomes included live birth and recurrent pregnancy loss. All indicators were validated as independent predictors through multivariate regression analysis prior to ROC analysis. METS-IR showed the highest predictive accuracy with an area under the curve (AUC) of 0.710, followed by TyG-BMI (AUC = 0.699), TG/HDL-C (AUC = 0.677), and TyG index (AUC = 0.646).

The predictive accuracy of four IR indicators (METS-IR, TyG-BMI, TG/HDL-C, TyG index) for pregnancy loss in RPL patients was assessed using ROC curve analysis, as shown in **Figure 3**. These indicators were validated as independent predictors through multivariate regression before ROC analysis. METS-IR demonstrated the highest predictive accuracy with an AUC of 0.710, compared to TyG-BMI (0.699), TG/HDL-C (0.677), and TyG index (0.646).

## 4 Discussion

We established the preconception period as critical for assessing the association between four IR indicators and recurrent pregnancy outcomes in women with RPL. Optimal cut-offs for predicting IR (HOMA-IR ≥ 2.5) in this Chinese cohort were: TG/HDL-C ≥ 1.91, TyG-BMI ≥ 144.68, and METS-IR ≥ 26.75. TG/HDL-C, TyG-BMI, and METS-IR demonstrated robust predictive profiles, whereas the TyG index exhibited fitted curve crossing, indicating greater diagnostic uncertainty. Our analyses further revealed that elevated preconception TyG-BMI and METS-IR were independently associated with increased RPL risk after full adjustment for confounders, with stable effect estimates across all models. By contrast, the TyG index showed no significant association in the fully adjusted model, consistent with its limited diagnostic utility as a standalone marker.

The TG/HDL-C mirrors lipid metabolism in IR (24). Pregnancy amplifies energy and nutrient demands, driving higher food intake and complex shifts in lipid profiles; gestational hormones further promote lipid storage (25). Consequently, the TG/HDL-C ratio is a weak predictor of subsequent pregnancy outcome in women with RPL. This limited discriminative power contrasts with earlier reports, a discrepancy attributable to differences in index properties and study populations (26).

The TyG index is calculated from fasting triglyceride and blood glucose levels and reflects the interaction between lipid and glucose metabolism. While the TyG index is recognized as a valid surrogate indicator of IR, consensus on its optimal threshold remains lacking across diverse geographical regions and populations (27). A critical factor contributing to the non-significant association of the TyG index is the unique physiological state of women during the conception period, which differs from that of the general population or non-reproductive cohorts. Conception women exhibit dynamic changes in sex hormones and metabolic homeostasis, which are closely linked to both IR and pregnancy outcomes. Estrogen, in particular, can regulate lipid metabolism and improve insulin sensitivity, which may weaken the correlation between the TyG index and IR in this specific population (28). Furthermore, since the index only reflects glycolipid metabolic disorders, excludes key covariates (e.g., body weight), and is confounded by age and BMI, its standalone use limits accurate assessment of the association between IR and pregnancy loss risk.

In contrast to single-dimensional indicators, the enhanced predictive efficacy of TyG-BMI and METS-IR for RPL can be linked to their comprehensive representation of systemic metabolic status. As a composite index, TyG-BMI amalgamates data on glycolipid metabolism and obesity, facilitating a more holistic evaluation of metabolic disturbances compared to single-dimensional indicators. This is consistent with contemporary research perspectives that identify systemic metabolic dysfunction as a fundamental pathophysiological basis for RPL (29). Combined disruptive effects of glycolipid metabolism dysfunction and obesity on reproductive endocrine function may directly reduce endometrial receptivity, disrupt maternal-fetal immune homeostasis, and provoke a hypercoagulable state or inflammatory responses-all of which are well-documented contributors to pregnancy failure (30). Similarly, METS-IR, another multi-dimensional metabolic index, integrates diverse metabolic information to effectively identify individuals with underlying IR, even in the absence of overt hyperglycemia. The central pathogenic mechanism of IR is characterized by impaired insulin signaling pathways, which encompass functional deficiencies in key components including insulin receptor substrate proteins and glucose transporters, ultimately associated with hyperinsulinemia and dysregulated inflammatory responses (31). As a comprehensive metabolic assessment tool, METS-IR enables precise identification of high-risk populations with IR. Although no evident glycemic abnormalities are observed, these subjects display immune homeostasis disruption at the maternal-fetal interface and diminished endometrial receptivity due to underlying metabolic dysfunction, thereby increasing the risk of pregnancy loss (32).

IR affects the immune balance at the maternal-fetal interface, increases pro-inflammatory cytokines, and disrupts endometrial functions, contributing to RPL (33). It is also associated with metabolic inflammation through triglycerides, worsening reproductive endocrine issues and raising RPL risk. The underlying biological mechanisms may involve IR-induced alterations in the Th1/Th2 immune balance at the maternal-fetal interface, which promote the secretion of pro-inflammatory cytokines and impair the expression of endometrial adhesion molecules and decidualization processes (34, 35).

TyG-BMI and METS-IR are readily measurable indicators that are well-suited for use in primary healthcare settings. Monitoring these parameters prior to pregnancy can facilitate the identification of patients at high risk for RPL. Interventions such as weight management, dietary modifications, and physical activity can ameliorate IR and subsequently reduce the risk of pregnancy loss (36). Lifestyle modifications constitute a fundamental strategy for addressing metabolic dysfunction, decreasing adiposity, and ameliorating hyperandrogenism, thereby supporting the perspective that a healthy lifestyle contributes to improved pregnancy outcomes (37).

This study has several limitations. First, we only used a single preconception measurement from a pregnancy loss cohort, failing to capture the temporal fluctuations of glucose, insulin, and lipid levels during the 18-month follow-up. Second, despite rigorous multivariable adjustment (including thyroid function, homocysteine, and 25-hydroxyvitamin D), collinearity screening, and comprehensive sensitivity/subgroup analyses, residual confounding from unmeasured factors (e.g., lifestyle, diet) cannot be fully excluded. Third, the exclusive inclusion of Chinese women limits the generalizability of our findings, which require validation in diverse ethnic populations. Fourth, although the HOMA-IR cutoff of > 2.5 was validated in RPL populations, it is mainly derived from Asian and Western data, and HOMA-IR distributions vary by ethnicity (9). Additionally, the geographically and ethnically homogeneous study population may limit the direct generalizability of the established cutoff values, necessitating prospective validation in larger, multi-ethnic, and geographically diverse international cohorts before clinical application.

## 5 Conclusion

Preconception IR indicators, the METS-IR demonstrated the highest predictive efficacy for subsequent pregnancy loss. These findings suggest that METS-IR may have substantial clinical utility in evaluating pregnancy loss risk, thereby facilitating the implementation of targeted preconception interventions.

## Data Availability

The original contributions presented in the study are included in the article/**Supplementary Material**. Further inquiries can be directed to the corresponding author.
