# Supplementary material for: Associations of four insulin resistance indicators with subsequent pregnancy outcomes in women with recurrent pregnancy loss
Source: Front Endocrinol (Lausanne). 2026 Apr 2;17:1767301. doi: 10.3389/fendo.2026.1767301 (PMC13082940; doi:10.3389/fendo.2026.1767301)
Supplement: Supplementary file 1 [file Table1.docx]

Contents

1. Multiple Imputation for missing .............................................................. 1

- 1. Table 1 Missing data patterns ........................................................ 1
  2. Table 2-6 Imputed Datasets ........................................................ 2
  3. Table 7 Pool estimates from multiple imputed data .......................7
  4. Table 8 Merged Regression Coefficients .........................................10

2. Collinearity check ......................................................................................12

- 1. Table 9 Collinearity check (VIF selection) ....................................12
  2. Table 10 Univariate analysis of covariates vs Y ............................14
  3. Table 11-14 Covariate selection for each exposure ....................... 16
  4. Table 15 Selected covariates .......................................................... 20

3.Threshold analysis ................................................................................... 21

3.1 Table 16 Threshold analysis for Insulin resistance .......................... 21

4. Sensitivity analyses ......................................................................................22

- 1. Table 17 Stratified analysis by age and pregnancy loss times .......22
  2. Table 18 Subgroup analysis (clinical intervention) .........................23

5.Study population characteristics ..................................................................24

- 1. Table 19 Recurrent pregnancy loss vs single pregnancy loss ..........24
  2. Table 20 Recurrent pregnancy loss vs Eligible Infertility ...............26
  3. Table 21 Excluded vs Eligible Participants .........................................28

1. Multiple Imputation for missing

We use random forest imputation (missForest package) to perform multiple imputation on missing data (generating 5 complete datasets), then build regression models for each imputed dataset, and finally pool the regression coefficients and standard errors of these 5 regression models.

Table 1 Missing data patterns:

| **Pattern** | **N** |
| --- | --- |
| nothing | 584 |
| HCY | 32 |
| 25(OH)D | 22 |
| 2hPG | 2 |
| FT3 | 1 |
| TSH | 2 |
| 25(OH)D, HCY | 48 |
| FT3, FT4 | 21 |
| FCP, 2hPG | 4 |
| 2hINS, 2hPG | 1 |
| FT3, HCY | 1 |
| FT3, FT4, TSH | 53 |
| TBIL, DBIL, IBIL | 1 |
| 2hPG, 2hINS, 2hCP | 11 |
| FT3, FT4, HCY | 1 |
| FT3, FCP, 2hCP | 1 |
| 25(OH)D, FCP, 2hCP | 1 |
| FT3, FT4, TSH, HCY | 12 |
| 25(OH)D, FT3, FT4, TSH | 4 |
| HCY, TBIL, DBIL, IBIL | 1 |
| HCY, 2hPG, 2hINS, 2hCP | 1 |
| 25(OH)D, FT3, FT4, TSH, HCY | 20 |
| FT3, FT4, HCY, FCP, 2hCP | 1 |
| TBIL, DBIL, IBIL, ALT, AST | 16 |
| 25(OH)D, HCY, 2hPG, 2hINS, 2hCP | 3 |
| FT3, FT4, TSH, 2hPG, 2hINS, 2hCP | 3 |
| 25(OH)D, TBIL, DBIL, IBIL, ALT, AST | 2 |
| HCY, TBIL, DBIL, IBIL, ALT, AST | 4 |
| 25(OH)D, HCY, TBIL, DBIL, IBIL, ALT, AST | 7 |
| FT3, FT4, TBIL, DBIL, IBIL, ALT, AST | 1 |
| FT3, FT4, TSH, TBIL, DBIL, IBIL, ALT, AST | 8 |
| 25(OH)D, FT3, FT4, TSH, HCY, 2hPG, 2hINS, 2hCP | 1 |
| FT3, FT4, TSH, HCY, TBIL, DBIL, IBIL, ALT, AST | 7 |
| 25(OH)D, FT3, FT4, TSH, TBIL, DBIL, IBIL, ALT, AST | 1 |
| 25(OH)D, FT3, FT4, TSH, HCY, TBIL, DBIL, IBIL, ALT, AST | 18 |
| 25(OH)D, FT3, FT4, TSH, HCY, 2hPG, 2hINS, 2hCP, TBIL, DBIL, IBIL, ALT, AST | 1 |

Table 2 Imputed Dataset 1

| **Exposure** | **Model I**  **OR95%CI *P*-value** | **Model II**  **OR95%CI *P*-value** | **Model III**  **OR95%CI *P*-value** |
| --- | --- | --- | --- |
| TG/HDL-C | 1.13 (1.04, 1.23) 0.003 | 1.11 (1.02, 1.21) 0.012 | 1.13 (1.03, 1.24) 0.007 |
| Low | Reference | Reference | Reference |
| Middle | 1.43 (1.00, 2.05) 0.052 | 1.38 (0.96, 1.98) 0.085 | 1.32 (0.90, 1.92) 0.151 |
| High | 1.44 (1.00, 2.06) 0.048 | 1.32 (0.92, 1.90) 0.137 | 1.28 (0.86, 1.91) 0.222 |
| TyG index | 1.32 (0.99, 1.75) 0.057 | 1.20 (0.89, 1.60) 0.224 | 1.11 (0.79, 1.57) 0.538 |
| Low | Reference | Reference | Reference |
| Middle | 0.96 (0.67, 1.37) 0.815 | 0.91 (0.64, 1.31) 0.631 | 0.88 (0.60, 1.28) 0.495 |
| High | 1.19 (0.84, 1.69) 0.334 | 1.06 (0.74, 1.52) 0.759 | 0.87 (0.57, 1.32) 0.505 |
| TyG-BMI | 1.02 (1.02, 1.03) 0.001 | 1.02 (1.02, 1.03) 0.001 | 1.02 (1.01, 1.03) 0.001 |
| Low | Reference | Reference | Reference |
| Middle | 0.97 (0.67, 1.40) 0.851 | 0.93 (0.64, 1.35) 0.701 | 0.92 (0.62, 1.35) 0.656 |
| High | 1.61 (1.13, 2.28) 0.008 | 1.45 (1.01, 2.07) 0.045 | 1.51 (1.01, 2.27) 0.045 |
| METS-IR | 1.10 (1.06, 1.14) 0.001 | 1.10 (1.06, 1.14) 0.001 | 1.07 (1.02, 1.11) 0.006 |
| Low | Reference | Reference | Reference |
| Middle | 0.96 (0.66, 1.38) 0.814 | 0.95 (0.65, 1.35) 0.728 | 0.98 (0.67, 1.43) 0.919 |
| High | 1.53 (1.07, 2.17) 0.018 | 1.47 (1.03, 2.09) 0.034 | 1.56 (1.06, 2.29) 0.024 |

Table 3 Imputed Dataset 2

| **Exposure** | **Model I**  **OR95%CI *P*-value** | **Model II**  **OR95%CI *P*-value** | **Model III**  **OR95%CI *P*-value** |
| --- | --- | --- | --- |
| TG/HDL-C | 1.13 (1.04, 1.23) 0.003 | 1.11 (1.02, 1.21) 0.012 | 1.13 (1.03, 1.24) 0.007 |
| Low | Reference | Reference | Reference |
| Middle | 1.43 (1.00, 2.05) 0.052 | 1.38 (0.96, 1.98) 0.085 | 1.31 (0.90, 1.91) 0.154 |
| High | 1.44 (1.00, 2.06) 0.048 | 1.32 (0.92, 1.90) 0.137 | 1.28 (0.86, 1.91) 0.222 |
| TyG index | 1.32 (0.99, 1.75) 0.057 | 1.20 (0.89, 1.60) 0.224 | 1.11 (0.79, 1.56) 0.546 |
| Low | Reference | Reference | Reference |
| Middle | 0.96 (0.67, 1.37) 0.815 | 0.91 (0.64, 1.31) 0.631 | 0.88 (0.60, 1.28) 0.494 |
| High | 1.19 (0.84, 1.69) 0.334 | 1.06 (0.74, 1.52) 0.759 | 0.88 (0.58, 1.34) 0.543 |
| TyG-BMI | 1.02 (1.02, 1.03) 0.001 | 1.02 (1.02, 1.03) 0.001 | 1.02 (1.01, 1.03) 0.001 |
| Low | Reference | Reference | Reference |
| Middle | 0.97 (0.67, 1.40) 0.851 | 0.93 (0.64, 1.35) 0.701 | 0.92 (0.62, 1.35) 0.655 |
| High | 1.61 (1.13, 2.28) 0.008 | 1.45 (1.01, 2.07) 0.045 | 1.51 (1.01, 2.27) 0.045 |
| METS-IR | 1.10 (1.06, 1.14) 0.001 | 1.10 (1.06, 1.14) 0.001 | 1.07 (1.02, 1.11) 0.006 |
| Low | Reference | Reference | Reference |
| Middle | 0.96 (0.66, 1.38) 0.814 | 0.92 (0.63, 1.32) 0.639 | 0.95 (0.65, 1.39) 0.804 |
| High | 1.53 (1.07, 2.17) 0.018 | 1.39 (1.03, 2.09) 0.037 | 1.47 (1.02, 2.17) 0.045 |

Table 4 Imputed Dataset 3

| **Exposure** | **Model I**  **OR95%CI *P*-value** | **Model II**  **OR95%CI *P*-value** | **Model III**  **OR95%CI *P*-value** |
| --- | --- | --- | --- |
| TG/HDL-C | 1.13 (1.04, 1.23) 0.003 | 1.11 (1.02, 1.21) 0.012 | 1.13 (1.03, 1.23) 0.013 |
| Low | Reference | Reference | Reference |
| Middle | 1.43 (1.00, 2.05) 0.052 | 1.38 (0.96, 1.98) 0.085 | 1.31 (0.90, 1.92) 0.142 |
| High | 1.44 (1.00, 2.06) 0.048 | 1.32 (0.92, 1.90) 0.137 | 1.24 (0.83, 1.85) 0.302 |
| TyG index | 1.32 (0.99, 1.75) 0.057 | 1.20 (0.89, 1.60) 0.224 | 1.12 (0.80, 1.58) 0.514 |
| Low | Reference | Reference | Reference |
| Middle | 0.96 (0.67, 1.37) 0.815 | 0.91 (0.64, 1.31) 0.631 | 0.87 (0.60, 1.27) 0.480 |
| High | 1.19 (0.84, 1.69) 0.334 | 1.06 (0.74, 1.52) 0.759 | 0.88 (0.58, 1.34) 0.550 |
| TyG-BMI | 1.02 (1.02, 1.03) 0.001 | 1.02 (1.01, 1.03) 0.001 | 1.02 (1.01, 1.03) 0.001 |
| Low | Reference | Reference | Reference |
| Middle | 0.97 (0.67, 1.40) 0.851 | 0.93 (0.64, 1.35) 0.701 | 0.92 (0.62, 1.35) 0.659 |
| High | 1.61 (1.13, 2.28) 0.008 | 1.45 (1.01, 2.07) 0.045 | 1.51 (1.01, 2.27) 0.045 |
| METS-IR | 1.10 (1.06, 1.14) 0.001 | 1.10 (1.06, 1.14) 0.001 | 1.07 (1.02, 1.11) 0.006 |
| Low | Reference | Reference | Reference |
| Middle | 0.96 (0.66, 1.38) 0.814 | 0.92 (0.63, 1.32) 0.639 | 0.95 (0.65, 1.39) 0.804 |
| High | 1.53 (1.07, 2.17) 0.018 | 1.39 (1.04, 1.99) 0.036 | 1.47 (1.02, 2.17) 0.045 |

Table 5 Imputed Dataset 4

| **Exposure** | **Model I**  **OR95%CI *P*-value** | **Model II**  **OR95%CI *P*-value** | **Model III**  **OR95%CI *P*-value** |
| --- | --- | --- | --- |
| TG/HDL-C | 1.13 (1.04, 1.23) 0.003 | 1.11 (1.02, 1.21) 0.012 | 1.13 (1.03, 1.23) 0.013 |
| Low | Reference | Reference | Reference |
| Middle | 1.43 (1.00, 2.05) 0.052 | 1.38 (0.96, 1.98) 0.085 | 1.31 (0.90, 1.92) 0.142 |
| High | 1.44 (1.00, 2.06) 0.048 | 1.32 (0.92, 1.90) 0.137 | 1.24 (0.83, 1.85) 0.302 |
| TyG index | 1.32 (0.99, 1.75) 0.057 | 1.20 (0.89, 1.60) 0.224 | 1.12 (0.80, 1.58) 0.514 |
| Low | Reference | Reference | Reference |
| Middle | 0.96 (0.67, 1.37) 0.815 | 0.91 (0.64, 1.31) 0.631 | 0.88 (0.60, 1.28) 0.503 |
| High | 1.19 (0.84, 1.69) 0.334 | 1.06 (0.74, 1.52) 0.759 | 0.88 (0.58, 1.34) 0.560 |
| TyG-BMI | 1.02 (1.02, 1.03) 0.001 | 1.02 (1.01, 1.03) 0.001 | 1.02 (1.01, 1.03) 0.001 |
| Low | Reference | Reference | Reference |
| Middle | 0.97 (0.67, 1.40) 0.851 | 0.93 (0.64, 1.35) 0.701 | 0.92 (0.62, 1.35) 0.666 |
| High | 1.61 (1.13, 2.28) 0.008 | 1.45 (1.01, 2.07) 0.045 | 1.55 (1.03, 2.32) 0.035 |
| METS-IR | 1.10 (1.06, 1.14) 0.001 | 1.10 (1.06, 1.14) 0.001 | 1.07 (1.02, 1.11) 0.006 |
| Low | Reference | Reference | Reference |
| Middle | 0.96 (0.66, 1.38) 0.814 | 0.92 (0.63, 1.32) 0.639 | 0.95 (0.65, 1.39) 0.804 |
| High | 1.53 (1.07, 2.17) 0.018 | 1.39 (1.04, 1.99) 0.036 | 1.48 (1.06, 2.27) 0.043 |

Table 6 Imputed Dataset 5

| **Exposure** | **Model I**  **OR95%CI *P*-value** | **Model II**  **OR95%CI *P*-value** | **Model III**  **OR95%CI *P*-value** |
| --- | --- | --- | --- |
| TG/HDL-C | 1.13 (1.04, 1.23) 0.003 | 1.11 (1.02, 1.21) 0.012 | 1.13 (1.03, 1.24) 0.007 |
| Low | Reference | Reference | Reference |
| Middle | 1.43 (1.00, 2.05) 0.052 | 1.38 (0.96, 1.98) 0.085 | 1.31 (0.90, 1.92) 0.151 |
| High | 1.44 (1.00, 2.06) 0.048 | 1.32 (0.92, 1.90) 0.137 | 1.28 (0.86, 1.90) 0.223 |
| TyG index | 1.32 (0.99, 1.75) 0.057 | 1.20 (0.89, 1.60) 0.224 | 1.12 (0.80, 1.58) 0.514 |
| Low | Reference | Reference | Reference |
| Middle | 0.96 (0.67, 1.37) 0.815 | 0.91 (0.64, 1.31) 0.631 | 0.88 (0.60, 1.28) 0.500 |
| High | 1.19 (0.84, 1.69) 0.334 | 1.06 (0.74, 1.52) 0.759 | 0.88 (0.58, 1.34) 0.554 |
| TyG-BMI | 1.02 (1.02, 1.03) 0.001 | 1.02 (1.01, 1.03) 0.001 | 1.02 (1.01, 1.03) 0.001 |
| Low | Reference | Reference | Reference |
| Middle | 0.97 (0.67, 1.40) 0.851 | 0.93 (0.64, 1.35) 0.701 | 0.92 (0.63, 1.35) 0.673 |
| High | 1.61 (1.13, 2.28) 0.008 | 1.45 (1.01, 2.07) 0.045 | 1.52 (1.01, 2.27) 0.044 |
| METS-IR | 1.10 (1.06, 1.14) 0.001 | 1.10 (1.06, 1.14) 0.001 | 1.07 (1.02, 1.11) 0.006 |
| Low | Reference | Reference | Reference |
| Middle | 0.96 (0.66, 1.38) 0.814 | 0.92 (0.63, 1.32) 0.639 | 0.95 (0.65, 1.39) 0.804 |
| High | 1.53 (1.07, 2.17) 0.018 | 1.39 (1.04, 2.02) 0.038 | 1.47 (1.05, 2.29) 0.045 |

Table 7 Pool estimates from multiple imputed data

Table 7.1 Pooled estimates (Model I)

| **Var.** | **Coefficients** | **Se** | **T** | ***P* value** |
| --- | --- | --- | --- | --- |
| TG/HDL-C | 1.130000 | 0.048469 | 23.313684 | 0.000000 |
| Low |  |  |  |  |
| Middle | 1.430000 | 0.267857 | 5.338667 | 0.000000 |
| High | 1.440000 | 0.270408 | 5.325283 | 0.000000 |
| TyG index | 1.320000 | 0.193878 | 6.808421 | 0.000000 |
| Low |  |  |  |  |
| Middle | 0.960000 | 0.178571 | 5.376000 | 0.000000 |
| High | 1.190000 | 0.216837 | 5.488000 | 0.000000 |
| TyG-BMI | 1.020000 | 0.002551 | 399.840000 | 0.000000 |
| Low |  |  |  |  |
| Middle | 0.970000 | 0.186224 | 5.208767 | 0.000000 |
| High | 1.610000 | 0.293367 | 5.488000 | 0.000000 |
| METS-IR | 1.100000 | 0.020408 | 53.900000 | 0.000000 |
| Low |  |  |  |  |
| Middle | 0.960000 | 0.183673 | 5.226667 | 0.000000 |
| High | 1.530000 | 0.280612 | 5.452364 | 0.000000 |

Table 7.2 Pooled estimates (Model II)

| **Var.** | **Coefficients** | **Se** | **T** | ***P* value** |
| --- | --- | --- | --- | --- |
| TG/HDL-C | 1.110000 | 0.048469 | 22.901053 | 0.000000 |
| Low |  |  |  |  |
| Middle | 1.380000 | 0.260204 | 5.303529 | 0.000000 |
| High | 1.320000 | 0.250000 | 5.280000 | 0.000000 |
| TyG index | 1.200000 | 0.181122 | 6.625352 | 0.000000 |
| Low |  |  |  |  |
| Middle | 0.910000 | 0.170918 | 5.324179 | 0.000000 |
| High | 1.060000 | 0.198980 | 5.327179 | 0.000000 |
| TyG-BMI | 1.020000 | 0.004269 | 238.950104 | 0.000000 |
| Low |  |  |  |  |
| Middle | 0.930000 | 0.181122 | 5.134648 | 0.000000 |
| High | 1.450000 | 0.270408 | 5.362264 | 0.000000 |
| METS-IR | 1.100000 | 0.020408 | 53.900000 | 0.000000 |
| Low |  |  |  |  |
| Middle | 0.926000 | 0.177043 | 5.230378 | 0.000000 |
| High | 1.406000 | 0.257917 | 5.451372 | 0.000000 |

Table 7.3 Pooled estimates (Model III)

| Var. | Coefficients | Se | T | P value |
| --- | --- | --- | --- | --- |
| TG/HDL-C | 1.130000 | 0.052566 | 21.496835 | 0.000000 |
| Low |  |  |  |  |
| Middle | 1.312000 | 0.259734 | 5.051314 | 0.000000 |
| High | 1.264000 | 0.265215 | 4.765947 | 0.000002 |
| TyG index | 1.116000 | 0.198548 | 5.620819 | 0.000000 |
| Low |  |  |  |  |
| Middle | 0.878000 | 0.173020 | 5.074558 | 0.000000 |
| High | 0.878000 | 0.193422 | 4.539303 | 0.000006 |
| TyG-BMI | 1.020000 | 0.005102 | 199.920000 | 0.000000 |
| Low |  |  |  |  |
| Middle | 0.920000 | 0.185717 | 4.953771 | 0.000001 |
| High | 1.520000 | 0.323438 | 4.699513 | 0.000003 |
| METS-IR | 1.070000 | 0.022959 | 46.604444 | 0.000000 |
| Low |  |  |  |  |
| Middle | 0.956000 | 0.190280 | 5.024163 | 0.000001 |
| High | 1.490000 | 0.307791 | 4.840952 | 0.000001 |

Table 8 Merged Regression Coefficients: Multiple Regression Results

| **Exposure** | **Model I**  **OR95%CI *P*-value** | **Model II**  **OR95%CI *P*-value** | **Model III**  **OR95%CI *P*-value** |
| --- | --- | --- | --- |
| TG/HDL-C | 1.13 (1.04, 1.23) 0.003 | 1.11 (1.02, 1.21) 0.012 | 1.13 (1.03, 1.24) 0.007 |
| Low | Reference | Reference | Reference |
| Middle | 1.43 (1.00, 2.05) 0.052 | 1.38 (0.96, 1.98) 0.085 | 1.31 (0.90, 1.92) 0.151 |
| High | 1.44 (1.00, 2.06) 0.048 | 1.32 (0.92, 1.90) 0.137 | 1.26 (0.86, 1.90) 0.223 |
| TyG index | 1.32 (0.99, 1.75) 0.057 | 1.20 (0.89, 1.60) 0.224 | 1.12 (0.80, 1.58) 0.514 |
| Low | Reference | Reference | Reference |
| Middle | 0.96 (0.67, 1.37) 0.815 | 0.91 (0.64, 1.31) 0.631 | 0.88 (0.60, 1.28) 0.500 |
| High | 1.19 (0.84, 1.69) 0.334 | 1.06 (0.74, 1.52) 0.759 | 0.88 (0.58, 1.34) 0.554 |
| TyG-BMI | 1.02 (1.02, 1.03) 0.001 | 1.02 (1.01, 1.03) 0.001 | 1.02 (1.01, 1.03) 0.001 |
| Low | Reference | Reference | Reference |
| Middle | 0.97 (0.67, 1.40) 0.851 | 0.93 (0.64, 1.35) 0.701 | 0.92 (0.63, 1.35) 0.673 |
| High | 1.61 (1.13, 2.28) 0.008 | 1.45 (1.01, 2.07) 0.045 | 1.52 (1.01, 2.27) 0.044 |
| METS-IR | 1.10 (1.06, 1.14) 0.001 | 1.10 (1.06, 1.14) 0.001 | 1.07 (1.02, 1.11) 0.006 |
| Low | Reference | Reference | Reference |
| Middle | 0.96 (0.66, 1.38) 0.814 | 0.93 (0.63, 1.32) 0.639 | 0.96 (0.65, 1.39) 0.804 |
| High | 1.53 (1.07, 2.17) 0.018 | 1.41 (1.04, 2.02) 0.038 | 1.49 (1.05, 2.29) 0.045 |

1. Collinearity check

Collinearity was evaluated by the variance inflation factor (VIF); variables with VIF≥10 were excluded, and covariates were retained if *P*＜0.10 or if they altered the main risk factor’s coefficient by >10% in the basic model.
Table 9 Collinearity check (VIF selection):

|  | **Step 1** | **Step 2** | **Step 3** | **Step 4** | **Step 5** |
| --- | --- | --- | --- | --- | --- |
| TyG index | 64.1 | 13.9 | 13.8 | 9.1 | 6.3 |
| TG/HDL-C | 8.9 | 8.8 | 8.8 | 5.6 | 5.3 |
| TyG-BMI | 487.8 | 77.5 | 77.5 | 77.5 | 25.6 |
| METS-IR | 88.1 | 87.7 | 87.7 | 87.7 | 27.3 |
| Age | 1.4 | 1.4 | 1.4 | 1.4 | 1.4 |
| BMI | 302.2 | NA | NA | NA | NA |
| Education | 1.1 | 1.1 | 1.1 | 1.1 | 1.1 |
| Race | 1.1 | 1.1 | 1.1 | 1.1 | 1.1 |
| Menstrual cycles | 1.1 | 1.1 | 1.1 | 1.1 | 1.1 |
| Pregnancy loss types | 1.2 | 1.2 | 1.2 | 1.2 | 1.2 |
| 25(OH)D | 1.1 | 1.1 | 1.1 | 1.1 | 1.1 |
| FT3 | 1.5 | 1.5 | 1.5 | 1.5 | 1.5 |
| FT4 | 1.5 | 1.5 | 1.5 | 1.5 | 1.5 |
| TSH | 1.1 | 1.1 | 1.1 | 1.1 | 1.1 |
| HCY | 1.1 | 1.1 | 1.1 | 1.1 | 1.1 |
| FPG | 2.4 | 2.4 | 2.4 | 2.2 | 2 |
| INS | 2.8 | 2.8 | 2.8 | 2.8 | 2.8 |
| FCP | 2.4 | 2.4 | 2.4 | 2.4 | 2.4 |
| 2hPG | 2.1 | 2.1 | 2.1 | 2.1 | 2.1 |
| 2hINS | 4.9 | 4.9 | 4.9 | 4.9 | 4.9 |
| 2hCP | 4.7 | 4.6 | 4.6 | 4.6 | 4.6 |
| TC | 10.7 | 10.6 | 10.6 | 10.2 | 7.4 |
| TG | 14 | 13.7 | 13.7 | NA | NA |
| HDL-C | 11.3 | 11.3 | 11.3 | 11.2 | NA |
| LDL-C | 8.8 | 8.8 | 8.7 | 8.3 | 5.8 |
| Cr | 1.2 | 1.2 | 1.2 | 1.2 | 1.2 |
| UA | 1.3 | 1.3 | 1.3 | 1.3 | 1.3 |
| TBIL | 28.2 | 28.1 | NA | NA | NA |
| DBIL | 5.8 | 5.8 | 1.2 | 1.2 | 1.2 |
| IBIL | 21.3 | 21.3 | 1.1 | 1.1 | 1.1 |
| ALT | 2.8 | 2.8 | 2.8 | 2.8 | 2.7 |
| AST | 2.6 | 2.6 | 2.6 | 2.6 | 2.6 |
| RPL.RCD | 1.1 | 1.1 | 1.1 | 1.1 | 1.1 |

Variables removed： BMI
Variables removed： TG
Variables removed： HDL-C
Variables removed： TBIL

Y= OUTCOME
Table 10 Univariate analysis of covariates vs Y

| **Covariates** | **N** | **term** | **beta** | **Se.** | **exp(beta)** | **95%CI Low** | **95%CI Upp** | ***P*.value** |
| --- | --- | --- | --- | --- | --- | --- | --- | --- |
| Age | 897 | Age | 0.0563 | 0.0176 | 1.0580 | 1.0221 | 1.0951 | 0.0014 |
| Education | 897 | Factor (Education)2 | -0.3414 | 0.3305 | 0.7108 | 0.3719 | 1.3586 | 0.3017 |
|  |  | Factor (Education)3 | -0.4956 | 0.3136 | 0.6092 | 0.3295 | 1.1264 | 0.1140 |
| Race | 897 | Factor (Race)2 | -0.0567 | 0.3048 | 0.9449 | 0.5199 | 1.7172 | 0.8525 |
|  |  | Factor (Race)3 | -0.3685 | 0.4083 | 0.6918 | 0.3107 | 1.5401 | 0.3668 |
| Menstrual cycles | 897 | Factor (Menstrual cycles)1 | 0.1862 | 0.2080 | 1.2046 | 0.8013 | 1.8110 | 0.3709 |
| Pregnancy loss types | 897 | Factor (Pregnancy loss types)2 | 0.1100 | 0.1752 | 1.1162 | 0.7918 | 1.5735 | 0.5302 |
| 25(OH)D (nmol/L) | 897 | 25(OH)D | -0.0032 | 0.0147 | 0.9968 | 0.9686 | 1.0258 | 0.8259 |
| FT3 (pmol/L) | 897 | FT3 | -0.3200 | 0.1302 | 0.7262 | 0.5626 | 0.9372 | 0.0140 |
| FT4 (pmol/L) | 897 | FT4 | 0.0004 | 0.0258 | 1.0004 | 0.9511 | 1.0522 | 0.9886 |
| TSH (mIU/L) | 897 | TSH | 0.0090 | 0.0237 | 1.0090 | 0.9632 | 1.0571 | 0.7045 |
| HCY (μmol/L) | 897 | HCY | -0.0101 | 0.0180 | 0.9900 | 0.9556 | 1.0255 | 0.5754 |
| FPG (mmol/L) | 897 | FPG | -0.0887 | 0.1029 | 0.9151 | 0.7479 | 1.1196 | 0.3885 |
| INS (IU/L) | 897 | INS | -0.0103 | 0.0109 | 0.9898 | 0.9688 | 1.0112 | 0.3460 |
| FCP (ng/ml) | 897 | FCP | 0.0260 | 0.0852 | 1.0264 | 0.8686 | 1.2128 | 0.7599 |
| 2hPG (mmol/L) | 897 | 2hPG | 0.0501 | 0.0388 | 1.0513 | 0.9744 | 1.1344 | 0.1967 |
| 2hINS (IU/L) | 897 | 2hINS | 0.0018 | 0.0018 | 1.0018 | 0.9983 | 1.0053 | 0.3261 |
| 2hCP (ng/ml) | 897 | 2hCP | 0.0397 | 0.0289 | 1.0405 | 0.9833 | 1.1010 | 0.1691 |
| TC (mmol/L) | 897 | TC | 0.0925 | 0.0976 | 1.0969 | 0.9059 | 1.3282 | 0.3435 |
| LDL-C (mmol/L) | 897 | LDL-C | 0.2254 | 0.1150 | 1.2528 | 0.9999 | 1.5696 | 0.0501 |
| Cr (μmol/L) | 897 | Cr | -0.0062 | 0.0093 | 0.9938 | 0.9759 | 1.0120 | 0.5022 |
| UA(μmol/L) | 897 | UA | 0.0015 | 0.0012 | 1.0015 | 0.9992 | 1.0038 | 0.2056 |
| DBIL (μmol/L) | 897 | DBIL | -0.0212 | 0.0381 | 0.9790 | 0.9087 | 1.0549 | 0.5779 |
| IBIL (μmol/L) | 897 | IBIL | 0.0079 | 0.0165 | 1.0080 | 0.9759 | 1.0411 | 0.6299 |
| ALT(U/L) | 897 | ALT | 0.0008 | 0.0050 | 1.0008 | 0.9910 | 1.0107 | 0.8705 |
| AST(U/L) | 897 | AST | -0.0048 | 0.0088 | 0.9952 | 0.9782 | 1.0125 | 0.5852 |
| RPL recoded | 897 | Factor (Previous pregnancy losses)3 | 0.1940 | 0.1751 | 1.2141 | 0.8614 | 1.7113 | 0.2678 |
|  |  | Factor (Previous pregnancy losses)4 | 0.6781 | 0.2224 | 1.9702 | 1.2741 | 3.0465 | 0.0023 |

Add covariates to basic model or remove it from full model, check coeff. of X
Table 11 X= TyG index

|  |  | **Basic model** | **Full model** |  |
| --- | --- | --- | --- | --- |
| Covariate | +/- term | TYG1 | TYG1 | Selected |
|  | Initial X coeff. | 0.2752 | 0.2615 |  |
| Age | Age | 0.1810 * | 0.3056 * | Yes |
| Education | Factor (Education) | 0.2816 | 0.2650 |  |
| Race | Factor (Race) | 0.2712 | 0.2742 |  |
| Menstrual cycles | Factor (Menstrual cycles) | 0.2694 | 0.2645 |  |
| Pregnancy loss types | Factor (Pregnancy loss types) | 0.2710 | 0.2581 |  |
| 25(OH)D (nmol/L) | 25(OH)D | 0.2746 | 0.2675 |  |
| FT3 (pmol/L) | FT3 | 0.2551 | 0.2604 |  |
| FT4 (pmol/L) | FT4 | 0.2752 | 0.2659 |  |
| TSH (mIU/L) | TSH | 0.2727 | 0.2637 |  |
| HCY (μmol/L) | HCY | 0.2703 | 0.2591 |  |
| FPG (mmol/L) | FPG | 0.3661 * | 0.1825 * | Yes |
| INS (IU/L) | INS | 0.3776 * | 0.2130 * | Yes |
| FCP (ng/ml) | FCP | 0.2965 | 0.2669 |  |
| 2hPG (mmol/L) | 2hPG | 0.2401 * | 0.2641 | Yes |
| 2hINS (IU/L) | 2hINS | 0.2562 | 0.2595 |  |
| 2hCP (ng/ml) | 2hCP | 0.2343 * | 0.2578 | Yes |
| TC (mmol/L) | TC | 0.2571 | 0.2056 * | Yes |
| LDL-C (mmol/L) | LDL-C | 0.2209 * | 0.2442 | Yes |
| Cr (μmol/L) | Cr | 0.2748 | 0.2703 |  |
| UA(μmol/L) | UA | 0.2499 | 0.2765 |  |
| DBIL (μmol/L) | DBIL | 0.2692 | 0.2655 |  |
| IBIL (μmol/L) | IBIL | 0.2784 | 0.2589 |  |
| ALT(U/L) | ALT | 0.2869 | 0.2688 |  |
| AST(U/L) | AST | 0.3011 | 0.2610 |  |
| RPL recoded | Factor (Previous pregnancy losses) | 0.2505 | 0.2852 |  |

* >10% change compare to initial X coeff.

Table 12 X= TG/HDL-C

|  |  | **Basic model** | **Full model** |  |
| --- | --- | --- | --- | --- |
| Covariate | +/- term | TGHDL1 | TGHDL1 | Selected |
|  | Initial X coeff. | 0.1256 | 0.1081 |  |
| AGE | Age | 0.1069 * | 0.1191 * | Yes |
| Education | Factor (Education) | 0.1253 | 0.1106 |  |
| Race | Factor (Race) | 0.1254 | 0.1101 |  |
| Menstrual cycles | Factor (Menstrual cycles) | 0.1244 | 0.1095 |  |
| Pregnancy loss types | Factor (Pregnancy loss types) | 0.1252 | 0.1085 |  |
| 25(OH)D (nmol/L) | 25(OH)D | 0.1254 | 0.1097 |  |
| FT3 (pmol/L) | FT3 | 0.1241 | 0.1054 |  |
| FT4 (pmol/L) | FT4 | 0.1257 | 0.1097 |  |
| TSH (mIU/L) | TSH | 0.1255 | 0.1093 |  |
| HCY (μmol/L) | HCY | 0.1249 | 0.1080 |  |
| FPG (mmol/L) | FPG | 0.1331 | 0.1070 |  |
| INS (IU/L) | INS | 0.1528 * | 0.0959 * | Yes |
| FCP (ng/ml) | FCP | 0.1319 | 0.1083 |  |
| 2hPG (mmol/L) | 2hPG | 0.1198 | 0.1091 |  |
| 2hINS (IU/L) | 2hINS | 0.1236 | 0.1074 |  |
| 2hCP (ng/ml) | 2hCP | 0.1194 | 0.1066 |  |
| TC (mmol/L) | TC | 0.1233 | 0.1154 |  |
| LDL-C (mmol/L) | LDL-C | 0.1173 | 0.1160 |  |
| Cr (μmol/L) | Cr | 0.1257 | 0.1117 |  |
| UA(μmol/L) | UA | 0.1198 | 0.1126 |  |
| DBIL (μmol/L) | DBIL | 0.1244 | 0.1088 |  |
| IBIL (μmol/L) | IBIL | 0.1273 | 0.1069 |  |
| ALT(U/L) | ALT | 0.1286 | 0.1086 |  |
| AST(U/L) | AST | 0.1332 | 0.1055 |  |
| RPL recoded | Factor (Previous pregnancy losses) | 0.1215 | 0.1135 |  |

* >10% change compare to initial X coeff.

Table 13 X= TyG-BMI

|  |  | **Basic model** | **Full model** |  |
| --- | --- | --- | --- | --- |
| Covariate | +/- term | TYGBMI1 | TYGBMI1 | Selected |
|  | Initial X coeff. | 0.0062 | 0.0056 |  |
| AGE | Age | 0.0049 * | 0.0065 * | Yes |
| Education | Factor (Education) | 0.0061 | 0.0060 |  |
| Race | Factor (Race) | 0.0064 | 0.0052 |  |
| Menstrual cycles | Factor (Menstrual cycles) | 0.0061 | 0.0057 |  |
| Pregnancy loss types | Factor (Pregnancy loss types) | 0.0062 | 0.0056 |  |
| 25(OH)D (nmol/L) | 25(OH)D | 0.0062 | 0.0057 |  |
| FT3 (pmol/L) | FT3 | 0.0059 | 0.0054 |  |
| FT4 (pmol/L) | FT4 | 0.0063 | 0.0054 |  |
| TSH (mIU/L) | TSH | 0.0062 | 0.0056 |  |
| HCY (μmol/L) | HCY | 0.0062 | 0.0055 |  |
| FPG (mmol/L) | FPG | 0.0072 * | 0.0050 * | Yes |
| INS (IU/L) | INS | 0.0092 * | 0.0039 * | Yes |
| FCP (ng/ml) | FCP | 0.0070 * | 0.0056 | Yes |
| 2hPG (mmol/L) | 2hPG | 0.0058 | 0.0057 |  |
| 2hINS (IU/L) | 2hINS | 0.0061 | 0.0055 |  |
| 2hCP (ng/ml) | 2hCP | 0.0058 | 0.0055 |  |
| TC (mmol/L) | TC | 0.0060 | 0.0057 |  |
| LDL-C (mmol/L) | LDL-C | 0.0054 * | 0.0063 * | Yes |
| Cr (μmol/L) | Cr | 0.0062 | 0.0058 |  |
| UA(μmol/L) | UA | 0.0058 | 0.0060 |  |
| DBIL (μmol/L) | DBIL | 0.0062 | 0.0056 |  |
| IBIL (μmol/L) | IBIL | 0.0064 | 0.0054 |  |
| ALT(U/L) | ALT | 0.0066 | 0.0057 |  |
| AST(U/L) | AST | 0.0066 | 0.0057 |  |
| RPL recoded | Factor (Previous pregnancy losses) | 0.0056 | 0.0064 * | Yes |

* >10% change compare to initial X coeff.

Table 14 X= METS-IR

|  |  | **Basic model** | **Full model** |  |
| --- | --- | --- | --- | --- |
| Covariate | +/- term | METSIR1 | METSIR1 | Selected |
|  | Initial X coeff. | 0.0347 | 0.0310 |  |
| AGE | Age | 0.0287 * | 0.0359 * | Yes |
| Education | Factor (Education) | 0.0338 | 0.0333 |  |
| Race | Factor (Race) | 0.0357 | 0.0284 |  |
| Menstrual cycles | Factor (Menstrual cycles) | 0.0342 | 0.0320 |  |
| Pregnancy loss types | Factor (Pregnancy loss types) | 0.0346 | 0.0312 |  |
| 25(OH)D (nmol/L) | 25(OH)D | 0.0347 | 0.0316 |  |
| FT3 (pmol/L) | FT3 | 0.0335 | 0.0299 |  |
| FT4 (pmol/L) | FT4 | 0.0349 | 0.0298 |  |
| TSH (mIU/L) | TSH | 0.0348 | 0.0315 |  |
| HCY (μmol/L) | HCY | 0.0345 | 0.0310 |  |
| FPG (mmol/L) | FPG | 0.0393 * | 0.0281 | Yes |
| INS (IU/L) | INS | 0.0510 * | 0.0212 * | Yes |
| FCP (ng/ml) | FCP | 0.0393 * | 0.0312 | Yes |
| 2hPG (mmol/L) | 2hPG | 0.0327 | 0.0311 |  |
| 2hINS (IU/L) | 2hINS | 0.0345 | 0.0306 |  |
| 2hCP (ng/ml) | 2hCP | 0.0328 | 0.0304 |  |
| TC (mmol/L) | TC | 0.0343 | 0.0383 * | Yes |
| LDL-C (mmol/L) | LDL-C | 0.0310 * | 0.0397 * | Yes |
| Cr (μmol/L) | Cr | 0.0349 | 0.0327 |  |
| UA(μmol/L) | UA | 0.0328 | 0.0337 |  |
| DBIL (μmol/L) | DBIL | 0.0344 | 0.0313 |  |
| IBIL (μmol/L) | IBIL | 0.0355 | 0.0302 |  |
| ALT(U/L) | ALT | 0.0364 | 0.0316 |  |
| AST(U/L) | AST | 0.0365 | 0.0316 |  |
| RPL recoded | Factor (Previous pregnancy losses) | 0.0325 | 0.0352 * | Yes |

* >10% change compare to initial X coeff.

Table 15 Selected covariates

| **Y** | **X** | **Selected based on criteria 1** | **Selected based on criteria 2** |
| --- | --- | --- | --- |
| OUTCOME | TyG index | Age FPG INS 2hPG 2hCP TC LDL-C | Age FT3 FPG INS 2hPG 2hCP TC LDL-C Previous pregnancy losses |
| OUTCOME | TG/HDL-C | Age INS | Age FT3 INS LDL-C Previous pregnancy losses |
| OUTCOME | TyG-BMI | Age FPG INS FCP LDL-C Previous pregnancy losses | Age FT3 FPG INS FCP LDL-C Previous pregnancy losses |
| OUTCOME | METS-IR | Age FPG INS FCP TC LDL-C Previous pregnancy losses | Age FT3 FPG INS FCP TC LDL-C Previous pregnancy losses |

Notes:
1. Criteria 1：add the covariate to basic model or remove it from full model, change X coeff. >10%.
2. Criteria 2：criteria 1 or the covariate P<0.1 in univariate model vs Y.

1. Threshold analysis

Table 16 For outcome: Insulin resistance

| **Exposure:** | **TyG index** | **TG/HDL-C** | **METS-IR** | **TyG-BMI** |
| --- | --- | --- | --- | --- |
| Model I |  |  |  |  |
| One line effect | 4.86 (3.48, 6.80) <0.0001 | 1.62 (1.43, 1.85) <0.0001 | 1.16 (1.12, 1.19) <0.0001 | 1.03 (1.02, 1.04) <0.0001 |
| Model II |  |  |  |  |
| Turning point(K) | 7.88 | 1.91 | 26.75 | 144.68 |
| < K effect 1 | 12.93 (2.05, 81.65) 0.0065 | 2.45 (1.67, 3.58) <0.0001 | 1.15 (1.11, 1.19) <0.0001 | 0.98 (0.90, 1.07) 0.6217 |
| > K effect 2 | 4.30 (2.88, 6.40) <0.0001 | 1.38 (1.15, 1.65) 0.0006 | 1.58 (0.94, 2.67) 0.0849 | 1.03 (1.02, 1.04) <0.0001 |
| effect 2 - 1 | 0.33 (0.04, 2.51) 0.2855 | 0.56 (0.34, 0.92) 0.0230 | 1.38 (0.81, 2.34) 0.2371 | 1.05 (0.96, 1.15) 0.2529 |
| Model fit value at K | -0.73 (-0.98, -0.48) | 0.04 (-0.20, 0.28) | 1.20 (0.85, 1.56) | -1.38 (-1.67, -1.09) |
| LRT test | 0.271 | 0.023 | 0.048 | 0.035 |

Results in table: β (95%CI) P value / OR (95%CI) P value
Outcome: Insulin resistance
Exposure: TyG index; TG/HDL-C; METS-IR; TyG-BMI

1. Sensitivity analyses

Table 17 Stratified analysis by age (≥30 years vs <30 years) and number of pregnancy losses (≥4 times vs <4 times)

| **Outcome** | **N** | **TyG index** | **TG/HDL-C** | **TyG-BMI** | **METS-IR** |
| --- | --- | --- | --- | --- | --- |
| AGE categorical |  |  |  |  |  |
| <30 years | 401 | 1.05 (0.66, 1.67) | 1.05 (0.91, 1.22) | 1.00 (1.00, 1.01) | 1.02 (0.98, 1.06) |
| >=30 years | 496 | 1.49 (1.04, 2.16) | 1.17 (1.06, 1.30) | 1.01 (1.00, 1.01) | 1.04 (1.01, 1.08) |
| RPL recoded |  |  |  |  |  |
| 2 times | 576 | 1.26 (0.88, 1.82) | 1.11 (1.00, 1.23) | 1.00 (1.00, 1.01) | 1.03 (0.99, 1.06) |
| 3 times | 219 | 1.52 (0.88, 2.61) | 1.20 (1.01, 1.44) | 1.01 (1.00, 1.02) | 1.05 (1.00, 1.10) |
| ≥4 times | 102 | 0.91 (0.38, 2.18) | 1.11 (0.91, 1.36) | 1.01 (0.99, 1.02) | 1.04 (0.97, 1.12) |

Subgroup analysis restricted to women who received treatment for insulin resistance. Table 18 The associations between the four insulin resistance surrogate indicators and subsequent pregnancy outcomes

| **Outcome** | **N** | **TyG index** | **TG/HDL-C** | **TyG-BMI** | **METS-IR** |
| --- | --- | --- | --- | --- | --- |
| Clinical intervention |  |  |  |  |  |
| No | 168 | 2.07 (1.06, 4.06) | 1.13 (0.98, 1.32) | 1.01 (1.00, 1.02) | 1.03 (0.98, 1.08) |
| Yes | 257 | 1.13 (0.67, 1.91) | 1.09 (0.95, 1.25) | 1.01 (1.00, 1.02) | 1.02 (0.97, 1.07) |

1. Characteristics of study population

Table 19 Characteristics of study population (Recurrent pregnancy loss and single pregnancy loss)

| **GROUP** | **Recurrent pregnancy loss** | **One pregnancy loss** | ***P*-value** |
| --- | --- | --- | --- |
| N | 897 | 637 |  |
| AGE | 30.44 ± 4.19 | 29.75 ± 3.88 | <0.001 |
| BMI (kg/m2) | 22.43 ± 3.00 | 22.36 ± 3.30 | 0.672 |
| 25(OH)D (nmol/L) | 12.87 ± 5.06 | 12.15 ± 4.95 | 0.010 |
| FT3 (pmol/L) | 5.19 ± 0.88 | 5.26 ± 1.55 | 0.317 |
| FT4 (pmol/L) | 16.14 ± 2.86 | 16.18 ± 3.73 | 0.823 |
| TSH (mIU/L) | 2.75 ± 2.96 | 2.70 ± 2.02 | 0.744 |
| HCY (μmol/L) | 11.35 ± 4.17 | 11.03 ± 3.97 | 0.159 |
| FPG (mmol/L) | 5.07 ± 0.82 | 4.98 ± 0.82 | 0.079 |
| INS (mIU/L) | 11.80 ± 6.98 | 11.81 ± 6.40 | 0.977 |
| FCP (ng/ml) | 1.50 ± 0.85 | 1.40 ± 0.63 | 0.024 |
| 2hPG (mmol/L) | 6.23 ± 1.83 | 6.11 ± 1.83 | 0.255 |
| 2hINS (mIU/L) | 51.35 ± 40.17 | 52.39 ± 43.55 | 0.663 |
| 2hCP (ng/ml) | 5.14 ± 2.49 | 4.96 ± 2.38 | 0.192 |
| TC (mmol/L) | 3.97 ± 0.75 | 3.91 ± 0.76 | 0.142 |
| TG (mmol/L) | 1.17 ± 0.69 | 1.18 ± 0.77 | 0.898 |
| HDL-C (mmol/L) | 1.36 ± 0.35 | 1.37 ± 0.34 | 0.436 |
| LDL-C (mmol/L) | 2.49 ± 0.64 | 2.45 ± 0.62 | 0.198 |
| HOMAIR | 2.72 ± 1.87 | 2.81 ± 2.34 | 0.437 |
| Cr (μmol/L) | 53.19 ± 7.99 | 53.53 ± 8.00 | 0.434 |
| UA(μmol/L) | 264.70 ± 62.96 | 263.34 ± 62.93 | 0.695 |
| TBIL (μmol/L) | 12.67 ± 5.10 | 11.98 ± 4.92 | 0.013 |
| DBIL (μmol/L) | 2.94 ± 2.00 | 2.94 ± 1.82 | 0.998 |
| IBIL (μmol/L) | 9.69 ± 4.44 | 9.04 ± 4.48 | 0.008 |
| ALT(U/L) | 19.21 ± 14.60 | 21.17 ± 18.28 | 0.027 |
| AST(U/L) | 22.55 ± 8.61 | 22.77 ± 9.21 | 0.651 |
| Education (n, %) |  |  | 0.069 |
| Primary School | 47 (5.24) | 25 (3.92) |  |
| High School | 245 (27.31) | 148 (23.23) |  |
| College | 605 (67.45) | 464 (72.84) |  |
| Race |  |  | 0.464 |
| Han nationality | 804 (89.63) | 583 (91.52) |  |
| Hui nationality | 57 (6.35) | 33 (5.18) |  |
| Other nationality | 36 (4.01) | 21 (3.30) |  |
| MENASTRUAL |  |  | <0.001 |
| Regular | 773 (86.18) | 506 (79.43) |  |
| Irregular | 124 (13.82) | 131 (20.57) |  |
| RPLTYPE |  |  | <0.001 |
| Primary | 698 (77.81) | 544 (85.40) |  |
| Secondary | 199 (22.19) | 93 (14.60) |  |

Note：Results in table: Mean+SD / N (%)

*P* value*: Kruskal Wallis Rank Test for continuous variables, Fisher Exact for categorical variables with Expects＜10.

Table 20 Characteristics of study population (Recurrent pregnancy loss and Eligible Infertility)

| **GROUP** | **Recurrent pregnancy loss** | **Infertility** | ***P*-value** |
| --- | --- | --- | --- |
| N | 897 | 563 |  |
| AGE | 30.44 ± 4.19 | 30.45 ± 4.66 | 0.130 |
| BMI (kg/m2) | 22.43 ± 3.00 | 22.57 ± 3.56 | 0.419 |
| 25(OH)D (nmol/L) | 12.87 ± 5.06 | 12.66 ± 5.37 | 0.465 |
| FT3 (pmol/L) | 5.19 ± 0.88 | 5.21 ± 0.69 | 0.762 |
| FT4 (pmol/L) | 16.14 ± 2.86 | 15.77 ± 2.69 | 0.018 |
| TSH (mIU/L) | 2.75 ± 2.96 | 2.88 ± 2.19 | 0.412 |
| HCY (μmol/L) | 12.35 ± 4.17 | 12.47 ± 4.71 | 0.071 |
| FPG (mmol/L) | 5.07 ± 0.82 | 5.04 ± 0.74 | 0.540 |
| INS | 11.80 ± 6.98 | 11.47 ± 6.79 | 0.391 |
| FCP (ng/ml) | 1.50 ± 0.85 | 1.43 ± 0.66 | 0.117 |
| 2hPG (mmol/L) | 6.23 ± 1.83 | 6.32 ± 1.69 | 0.998 |
| 2hINS | 51.35 ± 40.17 | 52.16 ± 39.30 | 0.666 |
| 2hCP (ng/ml) | 5.14 ± 2.49 | 4.93 ± 2.26 | 0.061 |
| TC (mmol/L) | 3.97 ± 0.75 | 3.92 ± 0.65 | 0.702 |
| TG (mmol/L) | 1.17 ± 0.69 | 1.28 ± 0.66 | 0.044 |
| HDL-C (mmol/L) | 1.36 ± 0.35 | 1.36 ± 0.37 | 0.830 |
| LDL-C (mmol/L) | 2.49 ± 0.64 | 2.60 ± 0.75 | 0.003 |
| HOMAIR | 2.72 ± 1.87 | 2.90 ± 2.40 | 0.137 |
| Cr (μmol/L) | 53.19 ± 7.99 | 53.95 ± 8.08 | 0.089 |
| UA(μmol/L) | 264.70 ± 62.96 | 271.39 ± 66.49 | 0.063 |
| TBIL (μmol/L) | 12.67 ± 5.10 | 13.29 ± 6.37 | 0.046 |
| DBIL (μmol/L) | 2.94 ± 2.00 | 2.96 ± 1.67 | 0.818 |
| IBIL (μmol/L) | 10.69 ± 4.44 | 10.75 ± 4.54 | 0.777 |
| ALT(U/L) | 19.21 ± 14.60 | 18.61 ± 15.68 | 0.467 |
| AST(U/L) | 22.55 ± 8.61 | 23.35 ± 9.78 | 0.114 |
| Education (n, %) |  |  | 0.049 |
| Primary School | 47 (5.24) | 44 (7.82) |  |
| High School | 245 (27.31) | 169 (30.02) |  |
| College | 605 (67.45) | 350 (62.17) |  |
| Race |  |  | 0.103 |
| Han nationality | 804 (89.63) | 438 (77.80) |  |
| Hui nationality | 57 (6.35) | 76 (13.50) |  |
| Other nationality | 36 (4.01) | 49 (8.70) |  |
| Menstrual cycles (n, %) |  |  | 0.296 |
| Regular | 773 (86.18) | 474 (84.19) |  |
| Irregular | 124 (13.82) | 89 (15.81) |  |
| Pregnancy loss types (n, %) |  |  | 0.014 |
| Primary | 698 (77.81) | 406 (72.11) |  |
| Secondary | 199 (22.19) | 157 (27.89) |  |

Note:Results in table: Mean+SD / N(%)
P value*: Kruskal Wallis Rank Test for continuous variables, Fisher Exact for categorical variables with Expects＜10.

Table 21 Characteristics of study population (Excluded and Eligible Participants)

| **Characteristic** | **Excluded Participants** | **Eligible Participants** | ***P*-value** |
| --- | --- | --- | --- |
| N | 745 | 897 |  |
| Age（years） | 30.88 ± 4.57 | 30.46 ± 4.19 | 0.053 |
| BMI (kg/m^2^) | 22.45 ± 3.55 | 22.43 ± 3.00 | 0.884 |
| 25(OH)D (nmol/L) | 12.48 ± 5.26 | 12.87 ± 5.06 | 0.141 |
| FT3 (pmol/L) | 5.21 ± 0.66 | 5.19 ± 0.88 | 0.665 |
| FT4 (pmol/L) | 15.74 ± 2.60 | 16.14 ± 2.86 | 0.006 |
| TSH (mIU/L) | 2.79 ± 2.05 | 2.75 ± 2.96 | 0.769 |
| HCY (μmol/L) | 12.01 ± 4.52 | 11.67 ± 4.33 | 0.286 |
| FPG (mmol/L) | 5.04 ± 0.71 | 5.07 ± 0.82 | 0.532 |
| INS (mIU/L) | 11.29 ± 6.60 | 11.80 ± 6.98 | 0.157 |
| FCP (ng/ml) | 1.41 ± 0.64 | 1.50 ± 0.85 | 0.022 |
| 2hPG (mmol/L) | 6.02 ± 1.72 | 6.04 ± 1.74 | 0.891 |
| 2hINS (mIU/L) | 47.86 ± 40.01 | 47.87 ± 39.91 | 0.992 |
| 2hCP (ng/ml) | 4.89 ± 2.38 | 4.90 ± 2.42 | 0.965 |
| TC (mmol/L) | 4.03 ± 0.79 | 4.02 ± 0.78 | 0.817 |
| TG (mmol/L) | 1.23 ± 1.25 | 1.17 ± 0.69 | 0.268 |
| HDL-C (mmol/L) | 1.37 ± 0.39 | 1.36 ± 0.35 | 0.426 |
| LDL-C (mmol/L) | 2.58 ± 0.72 | 2.53 ± 0.67 | 0.782 |
| HOMA-IR | 2.97 ± 2.93 | 2.72 ± 1.87 | 0.054 |
| Cr (μmol/L) | 53.91 ± 7.98 | 53.19 ± 7.99 | 0.089 |
| UA(μmol/L) | 270.45 ± 66.66 | 264.70 ± 62.96 | 0.094 |
| TBIL (μmol/L) | 13.24 ± 6.21 | 12.67 ± 5.10 | 0.056 |
| DBIL (μmol/L) | 2.70 ± 1.77 | 2.94 ± 2.00 | 0.019 |
| IBIL (μmol/L) | 10.55 ± 5.57 | 9.69 ± 4.44 | 0.001 |
| ALT(U/L) | 18.92 ± 15.68 | 19.21 ± 14.60 | 0.713 |
| AST(U/L) | 23.12 ± 9.48 | 22.55 ± 8.61 | 0.236 |
| Education (n, %) |  |  | 0.379 |
| Primary School | 50 (6.71%) | 47 (5.24%) |  |
| High School | 210 (28.19%) | 245 (27.31%) |  |
| College | 485 (65.10%) | 605 (67.45%) |  |
| Race (n, %) |  |  | 0.052 |
| Han nationality | 771 (85.95%) | 804 (89.63%) |  |
| Hui nationality | 73 (8.14%) | 57 (6.35%) |  |
| Other nationality | 53 (5.91%) | 36 (4.01%) |  |
| Menstrual cycles (n, %) |  |  | 0.397 |
| Regular | 631 (84.70%) | 773 (86.18%) |  |
| Irregular | 114 (15.30%) | 124 (13.82%) |  |
| Pregnancy loss types (n, %) |  |  | 0.102 |
| Primary | 554 (74.36%) | 698 (77.81%) |  |
| Secondary | 191 (25.64%) | 199 (22.19%) |  |

Note: Mean+SD / N (%)，*P* value: Kruskal Wallis Rank Test for continuous variables, Fisher Exact for categorical variables with Expects＜10.
